# Revisiting the taxonomy of *Dioclea* and related genera (Leguminosae, Papilionoideae), with new generic circumscriptions

**DOI:** 10.3897/phytokeys.164.55441

**Published:** 2020-10-21

**Authors:** Luciano Paganucci de Queiroz, Cristiane Snak

**Affiliations:** 1 Programa de Pós-Graduação em Botânica, Universidade Estadual de Feira de Santana, Av. Transnordestina s/n, Novo Horizonte, 44036-900, Feira de Santana, BA, Brazil Universidade Estadual de Feira de Santana Feira de Santana Brazil; 2 Departamento de Engenharia de Pesca e Ciências Biológicas, Universidade do Estado de Santa Catarina, Rua Cel. Fernandes Martins 270, Progresso, 88790-000, Laguna, Santa Catarina, Brazil Universidade do Estado de Santa Catarina Laguna Brazil

**Keywords:** *
Cleobulia
*, *
Cymbosema
*, Diocleae, Fabaceae, *
Luzonia
*, *
Macropsychanthus
*, phylogeny, recircumscription

## Abstract

The Dioclea clade comprises four genera and aproximately 60 species of the tribe Diocleae: *Cleobulia* (4 species), *Cymbosema* (1), *Dioclea* (ca. 50), *Luzonia* (1) and *Macropsychanthus* (3–4). *Dioclea* has been demonstrated to be a non-monophyletic genus, but low sampling in previous phylogenetic studies hampered the adoption of new taxonomic arrangements. We carried out densely sampled phylogenetic analyses of the Dioclea clade using molecular markers that had performed well in previous studies: the ITS and ETS nuclear ribosomal regions and the plastid *trnK/matK*. Our results support the maintenance of the genera *Cleobulia* and *Cymbosema* with their current circumscriptions, but confirmed the polyphyly of *Dioclea*, with its species falling into three different positions: (1) the puzzling species, *Dioclea paniculata*, was highly supported as a member of the Galactia clade; (2) Dioclea subg. Dioclea appeared as sister to a clade composed of *Cleobulia* and *Cymbosema*; and (3) the species of *Dioclea* subgenera *Pachylobium* and *Platylobium* composed a paraphyletic grade nesting the genera *Luzonia* and *Macropsychanthus*. We thus propose that the circumscription of *Dioclea* should be restricted to Dioclea subg. Dioclea, with 13 species and that the limits of *Macropsychanthus* should be widened to include the genus *Luzonia*, as well as the *Dioclea* subgenera *Pachylobium* and *Platylobium*, with 46 species. Taxonomic summaries, new combinations and synonyms are presented for all genera of the Dioclea clade. *Cleobulia and Cymbosema* were retained in their original circumscriptions. We presented an illustrated taxonomic conspectus of all genera of the Dioclea clade including 44 new combinations, one new name, ten new synonyms, two re-established holotypes, 38 lectotypes, two epitypes and one neotype.

## Introduction

The genus *Dioclea* Kunth is one of the most important groups of tropical rainforest lianas. It includes some of the largest plants in primary forests, which are capable of spreading over wide areas on the canopies of the highest trees, often at heights above 30 m. With approximately 50 species in its current circumscription, the genus is distributed throughout the humid tropics of the Americas, Africa, Asia and the Pacific Islands. *Dioclea* is included in Diocleae, a tribe of Papilionoid legumes with 14 genera and approximately 200 species (Queiroz et al. 2015). Together with four other small genera, it composes the Dioclea clade, a monophyletic lineage that includes the geographically restricted genera *Cleobulia* Mart. ex Benth. (four species from the Neotropics), *Cymbosema* Benth. (one Amazonian and Mesoamerican species), *Luzonia* Elmer (one species from the Philippines) and *Macropsychanthus* Harms (2–3 species from New Guinea and neighbouring islands) (Queiroz et al. 2015).

In addition to a woody, coarse lianescent habit, the genera of the Dioclea clade also share trifoliolate leaves with stipellate leaflets, a pseudoracemose inflorescence with woody multiflorous nodes, rather large and robust firm flowers, a pseudomonadelphous androecium (i.e. with the 10 stamens joined in a tube, but with the vexillary stamen free at the base, forming fenestration via two holes at the base of the staminal tube) and a fleshy and robust intrastaminal nectary disc. Their large flowers are mostly pollinated by large carpenter bees, but some species are adapted for bird pollination (Arroyo 1981; Franco 1995; Peçanha 2014). Most species have large fruits and large seeds with long and linear (or short and oblong) hilum (Lackey 1981; Maxwell and Taylor 2003; Queiroz et al. 2003) and disperse their seeds through autochory, but some species have buoyant sea-drifted seeds (Muir 1933; Armstrong 2001).

The Dioclea clade is one of three highly-supported major lineages of the tribe Diocleae, as revealed by a multilocus molecular phylogeny using the nuclear ITS/5.8S and ETS regions and the plastid *matK* gene and the *trnT-Y* region (Queiroz et al. 2015). Previous studies, based on either morphological (Maxwell and Taylor 2003; Queiroz et al. 2003) or molecular (nrITS) data with sparser sampling (Varela et al. 2004), suggested its existence, but with low support. None of the previous studies supported the monophyly of the genus *Dioclea* and, instead, it was recovered as a biphyletic group roughly corresponding to long-recognised infrageneric taxa: the species of Dioclea sect. Dioclea grouping with the New World genera *Cleobulia* and *Cymbosema* (Maxwell & Taylor 2003; Queiroz et al. 2003, 2015; Varela et al. 2004; Sede et al. 2009) and the species belonging to sections *Pachylobium* Benth., *Platylobium* Benth. and *Macrocarpon* Amshoff nesting the representatives of the Old World genus *Macropsycanthus* (Maxwell and Taylor 2003; Queiroz et al. 2015). More recently, we included a sequence of the plastid *matK* gene of *Luzonia purpurea* Elmer in a broader phylogenetic analysis of the Leguminosae and it appeared as a sister to *Macropsychanthus*, nested within the second lineage of *Dioclea*, but with low support (LPWG 2017).

The morphological recognition of the two major lineages that include the species of *Dioclea* can be traced back to Bentham (1837), who divided the genus into the sections *Dioclea* (as *Eudioclea*) and *Pachylobium*. He later added a third section, *Platylobium* (Bentham 1859). Those three sections were diagnosed by a combination of just a few morphological traits: sect. Dioclea with stipules not prolonged beyond their base, keel petals straight and erostrate, all anthers fertile and uniform, fruits elastically dehiscent and seeds with a linear hilum; sect. Platylobium sharing with sect. Dioclea non-prolonged stipules, but with the keel strongly incurved, anthers alternately fertile and sterile, fruits flat compressed and obovate with 2–3 seeds near the apex and seeds with a short and oblong hilum; and sect. Pachylobium sharing with sect. Platylobium flowers with an incurved and rostrate keel and the anthers alternately fertile and sterile, but with stipules prolonged beyond their base, fruits indehiscent or partially dehiscent and seeds with a linear hilum encircling more than half of the seed’s circumference.

The circumscriptions of Bentham’s sections became less clear with the discovery of some Amazonian species that combined the diagnostic features of different sections, as was the case with *Dioclea macrocarpa* Huber and *D. erecta* Hoehne, which have androecia typical of sect. Dioclea and seeds typical of sect. Platylobium. Amshoff (1939) then created sect. Macrocarpon to include the species of *Dioclea* with stipules not prolonged beyond their base, androecium with uniform anthers, fruits mostly oblong with 4–5 seeds evenly distributed along their length and seeds with a short, oblong hilum. Maxwell (2011) elevated those three sections created by Bentham to subgenera and included Amshoff’s sect. Macrocarpon into subg. Platylobium.

Despite the existence of phylogenetic studies focusing on the tribe Diocleae, there has been no re-appraisal of the taxonomy of the Dioclea clade incorporating those findings. We can speculate that the situation probably reflects the rather sparse sampling of taxa across the morphological and geographical ranges of the included genera. Here, we thus provide a re-assessment of the taxonomy of the Dioclea clade in light of robust and densely-sampled phylogenetic analyses. These analyses sought to: (1) test the previous findings of paraphyly of the genus *Dioclea* and its relationships with the remaining genera of the Dioclea clade; (2) re-examine the monophyly of the infrageneric groups of *Dioclea*; and, (3) provide a new generic classification that reflects the phylogenetic structure of the Dioclea clade.

## Materials and methods

Taxon sampling was designed to test the monophyly of the Dioclea clade of the tribe Diocleae as identified by Queiroz et al. (2015), to test the monophyly of its genera and to explore relationships between the genera. The sampling included 62 accessions corresponding to: one species of the monospecific *Cymbosema*, four species of *Cleobulia* (100% of all species in the genus), one species of the monospecific *Luzonia*, one species and two varieties of *Macropsychanthus* (50% of the species and 33% of all taxa) and 36 described species (+ six inedit) of *Dioclea* (60%). *Canavalia bonariensis* Lindl. (Canavalia clade), *Cratylia mollis* Mart. ex Benth. and *Collaea stenophylla* (Hook. & Arn.) Benth. (Galactia clade) were selected as outgroups for phylogenetic analyses in the tribe Diocleae and *Deguelia nitidula* (Benth.) A.M.G. Azevedo & R.A. Camargo and *Muellera obtusa* (Benth.) M.J. Silva & A.M.G. Azevedo (Millettieae) were selected as more remote outgroups to root the trees. A complete list of the vouchers associated with GenBank accessions are presented in Table 1.

**Table 1.** Voucher information and GenBank accession numbers for the DNA sequences used in this study. Original sequences are presented with an asterisk.

Taxon	Voucher	Locality	GenBank accession numbers		
			ITS	ETS	*trnK/matK*
**OUTGROUPS (Tribe Millettieae)**					
*Deguelia nitidula* (Benth.) A.M.G. Azevedo & R.A. Camargo	L.P. Queiroz 14503 (HUEFS)	Brazil, Bahia	*MT565565	KC779809	KC779548
*Muellera obtusa* (Benth.) M.J. Silva & A.M.G. Azevedo	L.P. Queiroz 13959 (HUEFS)	Brazil, Bahia	*MT565566	KC779808	KC779550
**TRIBE DIOCLEAE**					
**CANAVALIA CLADE**					
*Canavalia bonariensis* Lindl.	C. Snak 518 (HUEFS)	Brazil, Paraná	KT751426	KT751375	KT751472
**GALACTIA CLADE**					
*Collaea stenophylla* (Hook. & Arn.) Benth.	L.P. Queiroz 12460 (HUEFS)	Brazil, Rio Grande do Sul	KC779802	KC779908	KC779566
*Cratylia mollis* Mart. ex Benth.	L.P. Queiroz 8024 (HUEFS)	Brazil, Bahia	KC779675	KC779879	KC779568
**DIOCLEA CLADE**					
***Cleobulia* Mart. ex Benth.**					
*Cleobulia crassistyla* R.H. Maxwell	S. Ronán 12224 (E)	Mexico, Guerrero	KC779672	KC779817	*MT565534
*Cleobulia leiantha* Benth.	I.P. Miranda 37 (INPA)	Brazil, Pará		KC779818	
*Cleobulia multiflora* Mart. ex Benth.	P.C.N. Jesus 13 (HUEFS)	Brazil, Bahia	KC779673	KC779819	KC779564
*Cleobulia diocleoides* Benth.	L.P. Queiroz 16306 (HUEFS)	Brazil, Bahia	*MT565567	*MT565546	*MT565535
***Cymbosema* Benth.**					
Cymbosema roseum Benth.	D. Cardoso 2868 (HUEFS)	Brazil, Amazonas	KC779676	KC779816	KC779569
Cymbosema roseum Benth.	C. Snak 1211 (HUEFS)	Brazil, Pará	*MT565568	*MT565547	*MT565536
***Dioclea* Kunth**					
**Dioclea subgen. Dioclea**					
Dioclea aff. virgata	C. Snak 1233 (HUEFS)	Brazil, Pará	*MT565569	*MT565548	*MT565537
*Dioclea apurensis* Kunth	L.P. Queiroz 13044 (HUEFS)	Brazil, Pará	KC779677		
*Dioclea apurensis* Kunth	N. Costa 2312 (HUEFS)	Brazil, Pará		KC779828	
*Dioclea burkartii* R.H. Maxwell	R.C. Salas s.n. (CTES)	Argentina, Corrientes	KC779680	KC779830	KC779571
*Dioclea fimbriata* Huber	C. Snak 1223 (HUEFS)	Brazil, Pará	*MT565571	*MT565551	*MT565539
Dioclea guianensis var. guianensis Benth.	M. Sanchez s.n. (CIAT 9311)	Colombia, Vichada	KC779689		KC779575
Dioclea guianensis var. holtiana Pittier ex R.H. Maxwell	E. Ventura 2837 (MEXU)	Mexico, Chiapas	*MT565572	*MT565552	*MT565540
*Dioclea lasiophylla* Mart.ex Benth.	D. Cardoso 2324 (HUEFS)	Brazil, Bahia	KC779692	KC779832	KC779578
*Dioclea sericea* Kunth	R. Schultze-Kraft s.n. (CIAT 9578)	Colombia, Cauca	KC779715	KC779823	KC779588
*Dioclea ulei* ined.	E.H.G. Ule 7169 (L)	Brazil, Piauí	*MT565582		
*Dioclea vallensis* R.H. Maxwell	D.J. Belalcazar s.n. (CIAT 17892)	Colombia, Antioquia	KC779718	KC779824	KC779591
Dioclea virgata var. crenata R.H. Maxwell	R. Schultze-Kraft s.n. (CIAT 18631)	Brazil, Pará	KC779682	KC779831	KC779572
Dioclea virgata var. virgata (Rich.) Amshoff	D. Cardoso 2917 (HUEFS)	Brazil, Rondônia	KC779723	KC779827	KC779593
**Dioclea subgen. Pachylobium (Benth.) R.H. Maxwell**					
*Dioclea aurea* R.H. Maxwell	A. Gentry 17811 (MEXU)	Colombia, Chocó		*MT565549	
*Dioclea densiflora* Huber	L.P. Queiroz 15904 (HUEFS)	Brazil, Pará	*MT565570	*MT565550	*MT565538
*Dioclea edulis* Kuhlm.	L.P. Queiroz 15226 (HUEFS)	Brazil, Bahia	KC779683	KC779835	KC779573
*Dioclea glabra* Benth.	L.P. Queiroz 10381 (HUEFS)	Brazil, Mato Grosso	KC779684	KC779837	
*Dioclea grandiflora* Mart. ex Benth.	L.P. Queiroz 7325 (HUEFS)	Brazil, Bahia	KC779686	KC779839	KC779574
*Dioclea grandistipula* L.P. Queiroz	H.C. Lima 6634 (HUEFS)	Brazil, Rio de Janeiro	KC779688	KC779840	
*Dioclea latifolia* Benth.	C. van den Berg 1163 (HUEFS)	Brazil, Bahia	KC779696	KC779843	KC779579
*Dioclea malacocarpa* Ducke	L.P. Queiroz 13076 (HUEFS)	Brazil, Pará	KC779698	KC779845	
*Dioclea marginata* Benth.	L.P. Queiroz 9136 (HUEFS)	Brazil, Bahia	KC779700	KC779847	KC779581
*Dioclea megacarpa* Rolfe	L.P. Queiroz 10135 (HUEFS)	Brazil, Piauí	KC779701		
*Dioclea paraguariensis* Hassl.	Cabid s.n. (CTES)	Argentina, Corrientes	KC779702	KC779848	
*Dioclea pulchra* Moldenke	M. Sousa 11095 (MEXU)	Panama, Darién	*MT565575	*MT565557	*MT565542
*Dioclea reflexa* Hook. f.	C. van den Berg 1796 (HUEFS)	Venezuela, Bolívar	KC779706	KC779856	KC779583
*Dioclea rugosa* ined.	B.A. Krukoff 8433 (P)	Brazil, Amazonas	*MT565576		
*Dioclea ruschii* ined.	L.P. Queiroz 15254 (HUEFS)	Brazil, Espírito Santo	KC779717	KC779854	KC779590
*Dioclea schottii* Benth.	S. Buzato 28114 (UEC)	Brazil, São Paulo	KC779710	KC779852	
*Dioclea sclerocarpa* Ducke	L.P. Queiroz 15911 (HUEFS)	Brazil, Pará	*MT565577	*MT565558	*MT565543
*Dioclea ucayalina* Harms	A. Grijalva 310 (MEXU)	Ecuador, Napo	*MT565581	*MT565562	
*Dioclea violacea* Mart. ex Benth.	D. Cardoso 637 (HUEFS)	Brazil, Bahia	KC779721		
*Dioclea violacea* Mart. ex Benth.	L.P. Queiroz 10135 (HUEFS)	Brazil, Piauí		KC779855	KC779855
*Dioclea wilsonii* Standl.	L.P. Queiroz 4899 (HUEFS)	Brazil, São Paulo	KC779725	KC779857	KC779594
*Dioclea* sp. nov.	L.T. Colín 1209 (MEXU)	Honduras, El Paraíso	*MT565579	*MT565560	*MT565545
*Dioclea* sp. nov.	J. Stehman 4721 (BHCB)	Brazil, Espírito Santo	*MT565579	*MT565561	
**Dioclea subgen. Platylobium (Benth.) R.H. Maxwell**					
*Dioclea bicolor* Benth.	L.P. Queiroz 10523 (HUEFS)	Brazil, Mato Grosso	KC779679	KC779833	
*Dioclea coriacea* Benth.	L.P. Queiroz 14315 (HUEFS)	Brazil, Goiás	KC779681	KC779834	
*Dioclea huberi* Ducke	J. Revilla 728 (MEXU)	Peru, Loreto		*MT565553	
*Dioclea huberi* Ducke	R. Vasquez 21022 (NY)	Peru, Amazonas		*MT565554	
*Dioclea macrocarpa* Huber	L.P. Queiroz 13910 (HUEFS)	Brazil, Amazonas	KC779697	KC779844	KC779580
*Dioclea paniculata* Killip ex R.H. Maxwell	M. Nee 8911 (MEXU)	Panama, Canal Zone	*MT565573	*MT565555	*MT565541
*Dioclea paniculata* Killip ex R.H. Maxwell	F.W. Pennel 2829 (NY)	Colombia, Cundinamarca	*MT565574	*MT565556	
*Dioclea pygmaea* ined.	L.P. Queiroz 10246 (HUEFS)	Brazil, Bahia	KC779704	KC779849	KC779582
Dioclea rostrata var. lanata	R. Schultze-Kraft s.n. (CIAT 8541)	Brazil, Tocantins	KC779691	KC779841	KC779577
Dioclea rostrata var. rostrata Benth.	L.P. Queiroz 14788 (HUEFS)	Brazil, Piauí	KC779708	KC779850	
*Dioclea scabra* (Rich.) R.H. Maxwell	L.P. Queiroz 13897 (HUEFS)	Brazil, Amazonas	KC779709	KC779851	KC779584
*Dioclea* sp. nov.	R. Farias 399 (CEN)	Brazil, Tocantins	*MT565578	*MT565559	*MT565544
***Luzonia* Elmer**					
*Luzonia purpurea* Elmer	Soejarto 7967 (F)	Philippines, Luzon	*MT565583	*MT565563	KX652152
***Macropsychanthus* Harms ex K. Schumann & Lauterbach**					
Macropsychanthus lauterbachii Harms var. lauterbachii	M. Hopkins 1360 (K)	Papua New Guinea	KP262490		KP658375
Macropsychanthus lauterbachii var. hirsutus Verd.	A.N. Millar NGF13855 (L)	Papua New Guinea, Morobe	*MT565584	*MT565564	

The DNA regions used in this study are the same as those used by Queiroz et al. (2015): the plastid *trnK/matK* (the *matK* gene and partial flanking *trnK* introns) and ribosomal nuclear ETS (partial 3’ end of the External Transcribed Spacer) and ITS (5.8S and flanking Internal Transcribed Spacers 1 and 2) (Table 2).

**Table 2.** Sequences of the primers used for PCR amplification and sequencing, as well as PCR conditions.

DNA region	Primer name	Primer Sequence 5’–3’	Reference	PCR Conditions					
				Pre-melting	Denaturation (I)	Primer Annealing (II)	Primer Extension (III)	Cycles (I + II + III)	Final Extension
ETS	18S-IGS	GAGACAAGCATATGACTACTGGCAGGATCAACCAG	Baldwin and Markos (1998)	94 °C (3 min)	94 °C (1 min)	55 °C (1 min)	72 °C (1.5 min)	30	72 °C (7 min)
	ETS-Dio	GCTTGTGCATCGAACGGTTGG	Queiroz et al. (2015)						
ITS	17SE (F)	ACGAATTCATGGTCCGGTGAAGTGTTCG	Sun et al. (1994)	94 °C (3 min)	94 °C (1 min)	52 °C (40 s)	72 °C (2.5 min)	28	72 °C (7 min)
	26SE (R)	TAGAATTCCCCGGTTCGCTCGCCGTTAC	Sun et al. (1994)						
	5.8S	ACGACTCTCGGCAAC	Sun et al. (1994)						
	5.8R	GCGTGACGCCCAGGC	Sun et al. (1994)						
	SSF	GTCGTAACAAGGTTTCCGTAG	Kollipara et al. (1997)	Following manufacturer’s protocol for sequencing					
	LSR	GTTAGTTTCTTTTCCTCC	Kollipara et al. (1997)						
*trnK/ matK*	matK685F	GTATCGCACTATGTATTATTTGA	Wojciechowski et al. (2004)	94 °C (3 min)	94 °C (40 s)	55 °C (45 s)	72 °C (1 min)	36	72 °C (7 min)
	matK4La	CCTTCGATACTGGGTGAAAGAT	Wojciechowski et al. (2004)						
	matK1100L	TTCAGTGGTACGGAGTCAAATG	Wojciechowski et al. (2004)						
	matK4R	CATCTTTCACCCAGTAGCGAAG	Hu et al. (2000)						
	matK1932R	CAGACCGGCTTACTAATGGG	Hu et al. (2000)						
	trnK2R	CCCGGAACTAGTCGGATG	Wojciechowski et al. (2004)						

Total genomic DNA was extracted from silica gel-dried leaves using the 2× CTAB protocol of Doyle and Doyle (1987). For herbarium samples, DNA was extracted using the DNeasy Plant Mini Kit (QIAGEN GmbH, Hilden, Germany). PCR reactions were performed using the TopTaq Master Mix Kit (QIAGEN GmbH, Hilden, Germany) according to the manufacturer’s protocols, with a final volume of 10 µl. For herbarium samples, the PCR reactions also included 2 µl of TBT-PAR [trealose, bovine serum albumin (BSA), polysorbate-20 (Tween-20)] (Samarakoon et al. 2013) and, for ITS, they also included 0.2 µl of 99.5% DMSO (dimethyl sulphoxide) to avoid secondary conformations. Primers and PCR conditions are summarised in Table 2.

The PCR products were cleaned using 11% PEG (Paithankar and Prasad 1991) and then sequenced in both directions using the Big Dye Terminator v3.1 Cycle Sequencing Kit (Applied Biosystems, Austin, Texas, USA) according to the following protocol: a hot start followed by 3 min of initial denaturation at 96 °C, 30 cycles of 96 °C denaturation for 20 s, 50 °C annealing for 15 s and a 60 °C extension for 4 min. Sequencing products were cleaned using 80% isopropanol and 70% ethanol and analysed on a 3130×l Genetic Analyser (Applied Biosystems/HITACHI, Tokyo, Japan) at the Laboratório de Sistemática Molecular de Plantas of the Universidade Estadual de Feira de Santana (LAMOL/UEFS).

The original electropherograms were assembled into final sequences using the Geneious platform (Drummond et al. 2012). The sequences were automatically aligned in MUSCLE with default settings (Edgar 2004) and then checked using Geneious for manual adjustments. We carried out maximum parsimony (MP), maximum likelihood (ML) and Bayesian analyses for both individual and combined (nrITS, nrETS and *trnK/matK*) DNA datasets. Conflicts amongst datasets were evaluated by the incongruence length difference test (ILD; Farris et al. 1995), performed in PAUP v.4.0b10 (Swofford 2002) between nuclear regions and between the nuclear and plastid regions, using a heuristic search with 1000 replicates, random taxa-addition and tree bisection and reconnection (TBR) branch-swapping, saving 15 trees per replicate.

The search for the most parsimonious trees was carried out in PAUP v. 4.0b10 (Swofford 2002). Heuristic searches were made with 1000 random taxon-addition and tree bisection-reconnection (TBR) branch swapping, saving 15 trees per replicate. The trees saved in this first round were used as starting trees for a subsequent round of TBR swapping. All character state transformations were weighted equally and unordered (Fitch 1971). Non-parametric bootstrap resampling was used to estimate clade support (Felsenstein 1985), which was assessed through 2000 replicates (Hedges 1992; Müller 2005), simple taxon-addition and TBR algorithm, saving 15 trees per replicate. Only bootstrap percentages > 85% were considered as strong support (Kress et al. 2002).

Bayesian analyses were performed using MrBayes v.3.2.7a (Ronquist et al. 2012) in CIPRES Science Gateway v.3.3 (Miller et al. 2010). Nucleotide substitution models were selected using the Akaike Information Criterion (AIC) in MrModeltest v.2.3 (Nylander 2004) for each DNA region (Table 3). Two runs using the Metropolis-coupled MCMC (Markov Chain Monte Carlo) algorithm, each with four random-initiated chains (one ‘cold’ and three ‘heated’), involved 10 million generations and those were sampled every 1000 generations. The convergence of the runs was assessed by checking if the standard deviation of split frequencies reached a value below 0.01. The first 2500 trees of each run were excluded as burn-ins and the effective sample size (ESS) of all parameters was checked to verify if the values were > 200. The remaining trees were summarised into a majority-rule consensus tree including the posterior probabilities (PP) as branch support estimates. Only PP values ≥ 95 were considered as strong support (Erixon et al. 2003). *Deguelia nitidula* was chosen as the outgroup in the Bayesian analyses.

**Table 3.** Features of the DNA datasets used in this study, based on one of the most parsimonious trees from the combined parsimony analysis and nucleotide substitution models selected for Bayesian analyses. (bp = base pairs; CI = consistency index; RI = retention index; Best-fit model for the Bayesian analysis was selected by AIC in MrModeltest 2.3).

DNA region	N	Aligned length (bp)	Number variable sites		Number Potentially parsimony informative sites		Number of changes/ variable sites	Fitch tree length	CI	RI	Best-fit model
ETS region	55	439	**277**	**(63.10%)**	205	(46.70%)	2.40	666	0.62	**0.82**	GTR+G
ITS region	56	687	320	(46.58%)	239	(34.79%)	2.38	762	0.60	**0.81**	mixed
ITS1		278	**158**	**(56.83%)**	118	(42.45%)	2.44	385	0.60	0.79	SYM+G
5.8S		164	13	(7.93%)	10	(6.10%)	1.15	15	0.87	0.94	K80
ITS2		245	**149**	**(60.82%)**	111	(45.31%)	2.43	362	0.59	0.82	SYM+G
*trnK* introns	40	407	76	(18.67%)	43	(10.57%)	1.29	98	0.85	**0.90**	GTR+I+G
*matK* gene		1539	244	(15.85%)	149	(9.68%)	1.25	306	0.83	**0.86**	mixed
*matK* (1^st^ positions)		513	71	(13.84%)	44	(8.58%)	1.23	87	0.85	0.87	GTR+G
*matK* (2^st^ positions)		513	64	(12.48%)	39	(7.60%)	1.16	74	0.88	0.93	GTR
*matK* (3^st^ positions)		513	109	(21.25%)	66	(12.87%)	1.33	145	0.8	0.8	GTR+G
Combined (all data)	60	3072	917	(29.85%)	636	(20.70%)	1.73	1832	0.66	0.82	mixed

Maximum likelihood analyses were carried out using RAxML v.8.2.12 (Stamatakis 2014) in CIPRES Science Gateway v.3.3 (Miller et al. 2010) under a GTRGAMMA model, with the ‘-f a’ option (search for the best-scoring ML tree and a rapid bootstrap analysis) and 1000 bootstrap replicates. The MP strict consensus trees, ML trees and Bayesian 50% majority-rule consensus trees were visualised and partially edited in FigTree v.1.4.4 (Rambaut 2018).

## Results

We generated 51 new sequences for the Dioclea clade (19 of the nuclear ETS, 20 of the nuclear ITS and 12 of the plastid *trnK/matK*). The most variable dataset was ETS, followed by ITS and *trnK/matK*, respectively (Table 3). In terms of informativeness as measured by the retention index (RI) of each dataset, the ETS and the ITS performed similarly and slightly worse than *trnK/matK*, suggesting that part of the variation in the nuclear datasets are homoplasious (Table 3).

**Figure 1.**
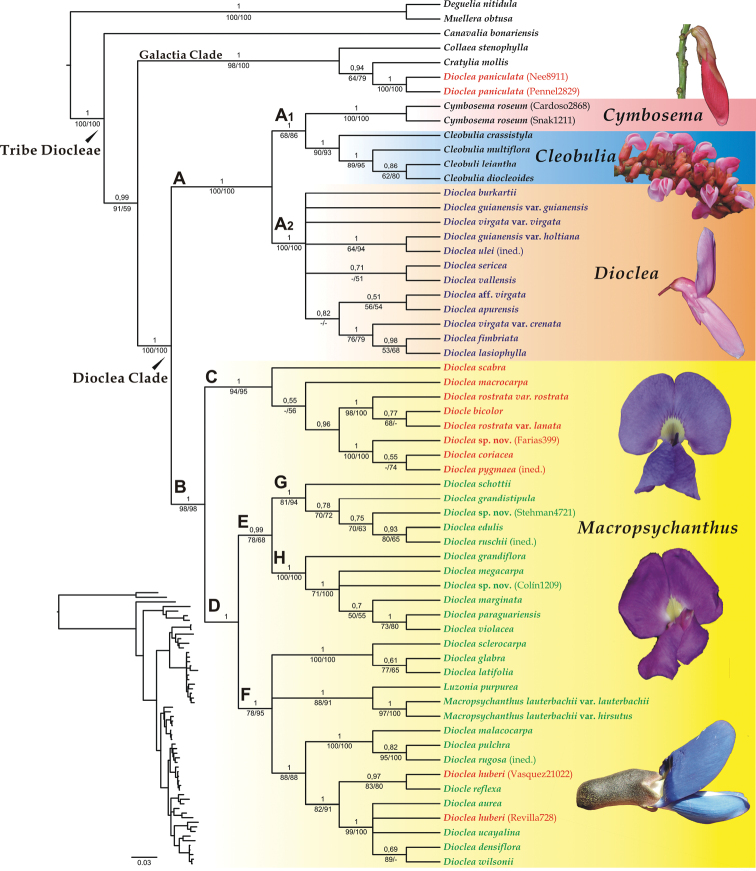
Majority rule Bayesian tree and respective phylogram of the Dioclea clade resulting from the combined nuclear (ETS, ITS) and plastid (*trnK/matK*) analysis. Bayesian posterior probabilities are reported above branches and parsimony (left) and maximum likelihood (right) bootstrap support values are reported below branches. Bootstrap values below 50% are represented by hyphens. The coloured boxes represent the four genera as circumscribed here – names in colour represent the subgenera of the genus *Dioclea* (according to Maxwell 2011): blue Dioclea subg. Dioclea, red Dioclea subg. Platylobium, green Dioclea subg. Pachylobium – pictures: *Cymbosema roseum* (from *Snak 1211*), *Cleobulia coccinea* (from *Queiroz 16029*), *Dioclea fimbriata* (from *Snak 1223*), *Macropsycanthus marginatus* (from *Queiroz 15225*), *Macropsycanthus lautherbachii* (from *Poulsen*, unvouchered).

The individual phylogenetic analyses demonstrated similar results in recovering the same major clades and presenting no strongly-supported incongruences (Suppl. material: Figs S1–S3). The ETS trees were better resolved than those from ITS and *trnK/matK* (Suppl. material: Figs S1–S3). However, resolution within the main clades of the tree (see below) varied amongst datasets and thus a better overall topology was obtained in the combined analyses. Since the ILD test indicated no incongruence between nuclear datasets (p = 0.3) or between nuclear and plastid datasets (p = 0.5), we performed combined analyses, which provided a better overall topology and higher support values for the nodes. Thus, we present and discuss the results from the combined analyses (Fig. 1).

The Dioclea clade, comprising the genera *Cleobulia*, *Cymbosema*, *Dioclea*, *Luzonia* and *Macropsychanthus*, was recovered as monophyletic with high support with the exclusion of *Dioclea paniculata* (Fig. 1). Two major clades were recovered: clade A, including the genera *Cleobulia* and *Cymbosema*, together with Dioclea subg. Dioclea; and clade B, including the genera *Luzonia* and *Macropsychanthus*, together with *Dioclea* subgs. *Pachylobium* and *Platylobium*. *Dioclea paniculata* (subg. Platylobium) grouped with the genera of the Galactia clade. The genus *Dioclea*, therefore, appears polyphyletic, while the rest of genera in Dioclea clade were resolved as monophyletic with high support.

Within clade A, *Cleobulia* and *Cymbosema* comprise a highly-supported clade, sister to Dioclea subg. Dioclea. Clade B presents two major clades: C and D. Clade C brings together species of Dioclea subg. Platylobium; and clade D includes species of Dioclea subg. Pachylobium together with *D. huberi* (subg. Platylobium) and nests the representatives of the genera *Luzonia* and *Macropsychanthus* within it.

The phylogenetic structure of Clade D shows some geographical and ecological trends in its two major clades, E and F. Clade E includes species mostly from eastern South America, including a subclade of species found in Atlantic rainforests (clade G), which is a sister to a clade of species found in seasonally dry forests (clade H). Clade F is mostly composed of species found in rainforests of the Amazon region, but includes the pantropical sea-drifted *D. reflexa* and *D. wilsonii*, as well as the Australasian genera *Luzonia* and *Macropsychanthus*.

## Discussion

### Criteria for genera circumscriptions

As the genus *Dioclea* has been demonstrated here (and elsewhere) as non-monophyletic (Varela et al. 2004; Maxwell and Taylor 2003; Queiroz et al. 2003, 2015; LPWG 2017), it should be reclassified to preserve the principle of monophyly. In deciding which monophyletic groups should be named, other principles besides monophyly should be taken into consideration to maximise support for monophyly, for phylogenetic information and for ease of identification (diagnosability; Backlund and Bremer 1998).

One possible taxonomic solution for resolving the non-monophyly of *Dioclea* would be to merge all of the genera of the Dioclea clade into a widely-circumscribed *Dioclea*, thus subsuming the genera *Cleobulia*, *Cymbosema*, *Luzonia* and *Macropsychanthus* within *Dioclea*. Although having high phylogenetic support, such a broadly-circumscribed genus would lack diagnosability with respect to other genera of the tribe Diocleae because it would result in a highly-heterogeneous genus, presenting variations in almost all of the characters used to diagnose the genera in the tribe Diocleae. At the other extreme, another taxonomic solution would be to split *Dioclea* into several smaller genera to preserve *Luzonia* and *Macropsychanthus* in their current circumscriptions (Queiroz et al. 2015; LPWG 2017). That option presents several drawbacks, however, as some of the smaller clades within clade B lack support and such narrowly-circumscribed genera would be highly redundant, as they would be defined by the same set of morphological traits and would therefore lack diagnosability.

We opted for the intermediate solution of splitting *Dioclea* into two genera corresponding to the two major clades, A2 and B. Clade A2 then corresponds to Dioclea subg. Dioclea and includes *D. sericea* Kunth, the type species of *Dioclea* and would, therefore, retain the name of the genus. Clade B then corresponds to the subgenera *Pachylobium* and *Platylobium*, plus the genera *Luzonia* and *Macropsychanthus*. The genus name *Macropsychanthus* has priority for this clade. Both of the proposed genera are monophyletic, have high phylogenetic support (Fig. 1) and are diagnosed by clear macromorphological characters – thus presenting low redundancy (as will be discussed below).

### The genus *Dioclea* with a narrower circumscription

The circumscription of Dioclea is restricted here to the subg. Dioclea (sensu Maxwell 2011) or sect. Dioclea (sensu Bentham 1837). This group had been recovered as monophyletic in most phylogenetic studies, based on either morphological (Queiroz et al. 2003) or DNA data (Varela et al. 2004; Queiroz et al. 2015). It has also been supported as sister to a clade composed of the genera *Cleobulia* and *Cymbosema* (Queiroz et al. 2015) or to the genus *Cymbosema* (Varela et al. 2004; *Cleobulia* was not sampled in that study).

*Dioclea*, as re-circumscribed here (hereafter *Dioclea* s.s.), *Cleobulia* and *Cymbosema* compose a clade of morphologically-similar genera, sharing fruits mostly oblong-linear, smaller than those of clade B (ranging from 9 to 13 cm long and 1.5 to 2 cm wide in clade A vs. 10 to 34 cm long and 3.5 to 6.5 cm wide in clade B), with flat and elastically-dehiscing valves. The seeds of those genera are also quite similar, being relatively small (ranging from 7 to 10 mm long, 4 to 7 mm wide and 2 to 4 mm thick in clade A vs. 20 to 35 mm long, 22 to 30 mm wide and 4 to 15 mm thick in clade B), with narrowly elliptic or oblong outlines, lenticular (i.e. slightly laterally compressed – elliptic in cross section), a linear hilum encircling almost half of the seed’s circumference and a hard, bony testa (mostly marbled). All species of those genera also share an androecium with ten fertile stamens (Table 4).

**Table 4.** Morphological comparison between the genera of the Dioclea clade as circumscribed here.

Characters	* Cleobulia *	* Cymbosema *	* Dioclea *	* Macropsychanthus *
**Habit**	Woody vines.	Woody vines.	Woody vines.	Mostly lianas, less frequently woody vines or shrubs.
**Stipules**	Basifixed.	Basifixed.	Basifixed.	Medifixed or basifixed.
**Inflorescence**	Axillary and with an arched axis.	Axillary and erect.	Axillary and erect.	Erect, mostly axillary but frequently cauliflorous.
**Inflorescence nodes**	Multiflorous and secundiflorous, sessile, globose.	Multiflorous and secundiflorous, sessile.	Multiflorous and secundiflorous, sessile.	Multiflorous and secundiflorous, stalked.
**Flower position**	Resupinate (i.e. the standard petal backwards and the set wing-keel petals upwards).	Not resupinate.	Not resupinate.	Not resupinate.
**Calyx**	Cylindrical, 4-lobed, the lobes shorter than the tube and of the same length; upper lobe entire and truncate (wider than longer).	Campanulate, 4-lobed, the lobes having almost the same length and mathching the length of the tube; upper lobe triangulate.	Campanulate, 4-lobed, the lobes having almost the same length and mathching the length of the tube; upper lobe triangulate.	Campanulate, rarely cylindrical, upper edge humped or convex, 4–5-lobed or deeply bilabiate, the lower lobe much longer than the remaining.
**Standard petal**	Pink or purple, pubescent towards the apex, ecallose and spreading or reflexed ca. 90°.	Bright red, pubescent towards the apex, ecallose and spreading.	Mostly purple, rarely reddish-purple, pubescent towards the apex, ecallose, reflexed.	Mostly purple, rarely blue, glabrous, 2-callose, reflexed.
**Wing petals**	Dwarf, much shorter than the other petals and sagittate.	As long as the keel.	As long as the keel.	About twice as long as the keel.
**Keel petals**	Upcurved ca. 90° with a truncate apex, upper margin smooth.	Straight, oblanceolate, apex rounded, upper margin smooth.	Straight, elliptic to obovate, apex rounded, upper margin upper margin dentate, serrate or fimbriate.	Triangular or semilunar, extending distally into a slender, obtuse or truncate beak.
**Androecium**	Pseudomonadelphous, the staminal tube pubescent at the base.	Diadelphous, the staminal sheath glabrous.	Pseudomonadelphous, the staminal tube glabrous.	Pseudomonadelphous, the staminal tube glabrous, rarely pubescent at the base.
**Anthers**	Monomorphic, all fertile.	Monomorphic, all fertile.	Monomorphic, all fertile.	Mostly dimorphic, 5 fertile alternating with 5 sterile or 6 fertile and 4 sterile or anthers monomorphic and all 10 fertile.
**Intrastaminal disc**	10-lobed.	Entire with a smooth rim.	Entire with a smooth rim.	10-dentate or 10-lobed.
**Gynoecium**	Ovary sessile, 6‒8-ovulate; style not swollen.	Ovary sessile, 5‒6-ovulate; style not swollen.	Ovary stipitate, 7‒15-ovulate; style not swollen.	Ovary sessile, 2‒5 (10)-ovulate; style swollen and frequently flattened distally.
**Fruit**	Oblong-linear, elastically dehiscent; thin ribs at the margins	Shortly oblong, elastically dehiscent, margins lacking ribs or wings	Oblong-linear, elastically dehiscent; upper margin provided with ribs or wings.	Various, cylindrical to flat compressed, indehiscent, passively dehiscent or elastically dehiscent; upper margin smooth or provided with ribs or wings.
**Seeds**	Lenticular with a linear hilum encircling ca. 1/2 of the seed circumference	Lenticular with a linear hilum encircling ca. 1/2 of the seed circumference	Lenticular with a linear hilum encircling ca. 1/2 of the seed circumference	Massive, orbicular or without a defined shape; hilum linear encircling 1/2 to 4/5 of the seed’s circumference or short and oblong.

*Cymbosema* was placed within *Dioclea* by Zamora (2000). It was found to be supported, however, as sister to *Cleobulia* and merging it into *Dioclea* would require that *Cleobulia* should likewise be placed into *Dioclea* s.s. *Cymbosema* can be differentiated from *Dioclea* s.s. by having diadelphous androecium, with the vexillary stamen free (vs. joined into a pseudomonadelphous androecium in *Dioclea* s.s.), petals bright red (vs. purple, white or reddish-purple), standard petal spreading (vs. reflexed > 90°), keel petals with margins entire (vs. upper margin serrate to fimbriate) and fruits short and oblong, ca. 2.5× longer than wide, with a long, downcurved persistent style and about 4 seeds (vs. fruits linear, ≥ 5× longer than wide, with 6–10 seeds). Maxwell (1970) reported the standard petal as spreading in *D. fimbriata* Huber and *D. macrantha* Huber, but the examination of more specimens than were available before evidenced that the flowers in anthesis of those species show a reflexed standard.

*Cleobulia* is quite distinct from *Dioclea* s.s. and *Cymbosema* in terms of flower and fruit traits. The flowers of *Cleobulia* are functionally resupinate due to the downcurved inflorescence rachis and show dwarf wings of less than half of the keel length that barely exceed the calyx (vs. wings and keel petals ± the same size in *Dioclea* s.s. and *Cymbosema*), a strongly upcurved keel bent ca. 90° (vs. keel straight), short calyx lobes with the upper ones broad and emarginate (vs. all calyx lobes triangulate and acute) and the base of the androecium pubescent (vs. androecium glabrous). The fruits of *Cleobulia* lack the distinct ribs (or wings) close to the upper suture that are characteristic of *Dioclea* s.s. fruits (Maxwell 1977).

With the exclusion of the species of the subgenera *Pachylobium* and *Platylobium*, *Dioclea* s.s. can be diagnosed by having the standard petal ecallose and pubescent towards the apex on the outer surface, wing and keel petals approximately the same length, keel petals straight with rounded apices and serrate to fimbriate upper margins, fruits oblong-linear with flat and elastically dehiscent woody valves, seeds 6–10, lenticular, with a linear hilum encircling almost half of the seed’s circumference.

### The genus *Macropsychanthus* with a broader circumscription

*Macropsychanthus*, in its original circumscription (Harms 1900; Verdcourt 1978, 1979), included three species from Malesia. Its circumscription is broadened here to include *Luzonia*, Dioclea subg. Pachylobium and Dioclea subg. Platylobium.

*Macropsychanthus* was usually compared to Dioclea subg. Pachylobium, with the major distinguishing feature being an androecium with ten fertile stamens in *Macropsychanthus*, vs. five fertile anthers alternating with five reduced and vestigial sterile anthers in Dioclea subg. Pachylobium (Harms 1900; Maxwell 1969, 2011; Verdcourt 1978, 1979). However, some species of Dioclea sect. Pachylobium present six fertile and four sterile stamens [e.g. *Dioclea hexandra* (Ralph) Mabb.] or all ten stamens fertile (e.g. *Dioclea umbrina* Elmer), thus making a morphological bridge with the Malesian *Macropsychanthus*.

In their original circumscriptions, both *Luzonia* and *Macropsychanthus* have distinctive calyx morphologies. *Luzonia* (sensu Elmer 1907) has a very distinctive calyx, with the lobes joined into two deeply separate, entire and obtuse lips. *Macropsychanthus* (sensu Harms 1900) has a cylindrical calyx with five subequal and obtuse teeth. *Dioclea* subgenera *Pachylobium* and *Platylobium* typically have a 4-lobed campanulate calyx, with the upper lobe shorter and broader than the others, with the lower lobe longer, upcurved and long acuminate.

The highly-supported clade C corresponds to Dioclea subg. Platylobium, as defined by Maxwell (2011), including both sections *Platylobium* and *Macrocarpon* (but with the exclusion of *D. huberi*, which appeared nested in clade D). A clade, composed of taxa of subg. Platylobium, was recovered only in analyses using molecular data (Queiroz et al. 2015); in analyses using morphological data, the taxa belonging to that subgenus comprised a paraphyletic grade nesting the representatives of Dioclea subg. Pachylobium (Queiroz et al. 2003), as well as the genera *Luzonia* and *Macropsychanthus* (Maxwell and Taylor 2003). The enigmatic species *Dioclea paniculata* Killip ex R.H. Maxwell, tentatively placed in subg. Platylobium by Maxwell (1978), appeared more closely related to the Galactia clade (and its phylogenetic and taxonomic position will be addressed in another article).

Thus, in the new circumscription presented here, *Macropsychanthus* is polymorphic in both androecium and calyx traits, but can be diagnosed by woody and robust pseudoracemes with the peduncle up to 1.5 cm thick, inflorescence nodosities stalked and secundiflorous, calyx with a humped or convex tube on the upper side, standard petal glabrous and bicallose towards the blade base, keel petals strongly upcurved, intrastaminal disc 10-lobed, ovary sessile and large fruits and seeds.

## Taxonomic treatment

### Key to the genera of the *Dioclea* clade

**Table:** 

1	Flowers with petals entirely glabrous; seeds 13–50+ mm long and 3–40+ mm wide with circular, squarish, ovate or elliptic outlines (if ovate or elliptic, then flat compressed, not biconvex), either with a short and oblong or long and linear hilum (then encircling 1/2 to 4/5 of the seed’s circumference)	*** Macropsychanthus ***
–	Flowers with the standard petal pubescent towards the apex on the outer surface; seeds up to 14 mm long and 3 mm wide with elliptic outlines, lenticular (biconvex) and with a linear hilum encircling ca. 1/2 of the seed’s circumference;	**2**
2	Flowers resupinate because of the arching inflorescence; wing petals dwarf, much shorter than the standard and keel petals; keel petals upcurved with truncate apices; staminal tube pubescent at the base; upper calyx lobe broad, usually widely emarginate; fruits without ribs or wings near or at the upper margin	*** Cleobulia ***
–	Flowers not resupinate, wing petals not dwarf, approximately the same (or half of the) length of the keel; keel petals straight with rounded apices; androecium glabrous; upper calyx lobe triangulate and acute; fruits with the upper margin ribbed or narrowly winged	**3**
3	Flowers with the vexillary stamen free, the androecium consequently diadelphous; standard petal bright red, usually spreading; fruit broadly oblong with ca. 4 seeds and a long, downward rostrum	*** Cymbosema ***
–	Flowers with the vexillary stamen fused with the staminal sheath in the middle, the androecium then pseudomonadelphous; standard petal purple, rarely withish-purple or reddish-purple, reflexed; fruit linear with (6)10–12 seeds and a shortly apiculate apex	*** Dioclea ***

### Conspectus of the *Dioclea* clade with new classification including new combinations, synonyms and typifications

#### 1. Dioclea

Taxon classificationPlantaeFabalesFabaceae

Kunth, Nov. Gen. Sp. (quarto ed.) 6: 437. 1823 [Sept. 1824].

1D40AC02-EA5A-5AEC-8CC2-81995B6EF909

Hymenospron Spreng., Syst. Veg. [Sprengel] 4(2): 283. 1827. Type: Hymenospron apurense (Kunth) Spreng. [≡Dioclea apurensis Kunth].
Dioclea Kunth sect. Dioclea [‘Eudioclea’] in Benth., Comm. Legum. Gen. 2: 69. 1837.
Crepidotropis Walp., Linnaea 14: 296. 1840. Type: Crepidotropis brasiliensis Walp. [= Dioclea virgata (Rich.) Amshoff].
Dioclea Kunth subg. Dioclea in R.H. Maxwell, Novon 21(2): 227. 2011.
Dioclea Kunth ser. Dioclea in R.H. Maxwell, Novon 21(2): 227. 2011.
Dioclea ser. Virgatae R.H. Maxwell, Novon 21(2): 229. 2011. Type: Dioclea virgata (Rich.) Amshoff.

##### Type.

[lectotype, designated by Britton and Wilson (1924)]. *Dioclea sericea* Kunth.

##### Description.

**Figure 2.**
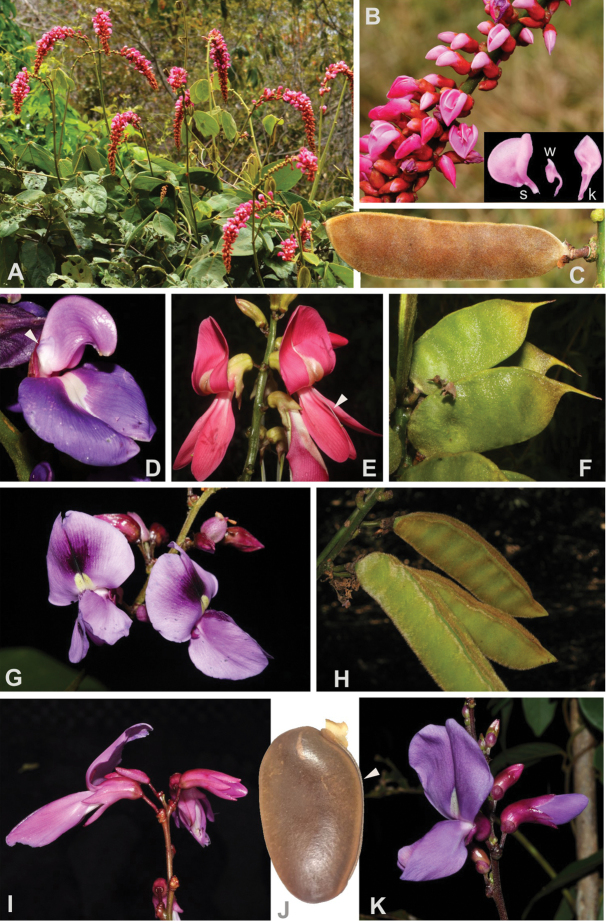
Representatives of the clade A. *Cleobulia coccinea* (Mart. ex Benth.) L.P. Queiroz **A** flowering vine showing the arcuate inflorescences **B** detail of the inflorescence showing resupinate flowers; the inset highlights the wing petals (w) much shorter than the standard (s) and keel petals (k) **C** fruit (from *Queiroz 16029*). *Cleobulia diocleoides* Benth. **D** a resupinate flower showing the reduced wing (from *Queiroz 16036*). *Cymbosema roseum* Benth. **E** part of the inflorescence showing the bird pollinated flowers and the free adaxial stamen (arrow) **F** immature fruits showing the characteristic broad oblong fruit body and the long beak (from *Cardoso2868*). *Dioclea virgata* (Rich.) Amshoff **G** flowers (from *Cardoso 2374*) **H** fruits (from *Cardoso 2100*). *Dioclea fimbriata* Huber **I** flowers (from *Snak 1223*). *Dioclea burkartii* R.H. Maxwell **J** a seed showing the marbled testa and the elongate hilum encircling about half of its circumpherence (arrow; from *Snak 826*). *Dioclea apurensis***K** flowers (from *Queiroz 13035*). Photos **A–D**, **J–K**: L.P. Queiroz; **E–H**: D. Cardoso; **I**: C. Snak.

Woody vines along forest edges, trailing or shrubby in open habitats. **Stipules** basifixed, not prolonged beyond their bases. **Leaves** pinnately trifoliolate, stipellate, leaf rachis short, mostly < 5 mm long. **Inflorescence** an erect pseudoraceme, nodes multiflorous, woody, sessile, secundiflorous; bracteoles chartaceous or membranous. **Flowers** with calyx chartaceous, campanulate, the four lobes having almost the same length, upper lobe entire, triangulate, obtuse or acute, the other three lobes triangulate, acute, the lower lobe as long as the upper lobe; petals membranous, mostly purple, rarely withish-purple or reddish-purple, standard petal reflexed, ecallose, but slightly thickened near the base, provided with two basal and reflexed auricles, pubescent towards the apex on the outer surface, wing petals as long as the keel, oblong to obovate, provided with a basal spur on the upper margin, keel petals straight, elliptic to obovate, upper margin dentate, serrate or fimbriate; androecium pseudomonadelphous, the 10 stamens joined into a tube but the filament of the vexillary stamen free at the base, anthers monomorphic, all 10 stamens fertile; intrastaminal nectary disc entire, collar-shape; pistil sigmoid, ovary mostly 7‒15-ovulate, stipitate, style not swollen. **Fruits** linear, mostly 5× longer than wide, up to 2.5 cm wide, elastically dehiscent, the thin woody valves explosively twisting to release the seeds, upper margin straight and provided with a longitudinal rib or wing to each side of the suture. **Seeds** small, up to 14 mm long and 8 mm wide, lenticular (slightly biconvex); testa hard (bony), smooth, mostly mottled; hilum linear, encircling almost half of the seed’s circumference (Fig. 2G–K).

##### Discussion.

*Dioclea* was described by Kunth (1823 [1824]) with two new species based on specimens collected by Humboldt and Bonpland: *D. apurensis*, from a depauperate fruiting specimen and *D. sericea*, with four flowering specimens and illustrated in plate 576. *Dioclea sericea* was selected as the type for the genus by Britton and Wilson (1924).

A few months after Kunth’s publication, Sprengel (1825) used the name *Dioclea* Spreng. for a genus of Boraginaceae. Later, Sprengel (1827) created the genus *Hymenospron* to which he transferred both of Kunth’s species, together with a species currently ascribed to *Galactia* [*G. rubra* (Jacq.) Urb.]. *Dioclea* Spreng. is a later homonym in relation to *Dioclea* Kunth and thus illegitimate. *Hymenospron* Spreng. is a superfluous name with respect to *Dioclea* Kunth. The genus *Crepidotropis* was created by Walpers (1840) with just one species (*C. brasiliensis*) that is conspecific with *Dioclea virgata* (Rich.) Amshoff.

The genus *Dioclea* was named after Diocles of Carystus, a Greek philosopher from the 3rd century BC., probably because he associated the word ‘beans’ with the genus *Dolichos* L., which, in its original circumscription, included species now ascribed to *Dioclea* (Candolle, 1825: 379‒380).

*Dioclea* is diagnosed by the combination of flowers with a pseudomonadelphous androecium, standard petal reflexed and pubescent towards the apex, fruits with an oblong-linear, flat compressed body and explosive dehiscence and seeds elliptic-oblong, lenticular, with a long and linear hilum encircling about half of their circumference.

As circumscribed here, *Dioclea* includes 13 species from the tropical Americas, ranging from coastal central Mexico to northern Argentina and Paraguay. *Dioclea virgata* was introduced into the Old World and became a garden escape plant in Malaysia, Borneo and Ethiopia (Maxwell 1969; Adema 1998).

#### 1.1. Dioclea albiflora

Taxon classificationPlantaeFabalesFabaceae

R.S. Cowan, Mem. New York Bot. Gard. 10(1): 150. 1958.

A061ACE5-3619-55B9-91F4-C42068EE02E0

##### Type.

Venezuela, Bolivar, Piedra Marimare, *Wurdack & Monachino 39980* (holotype: NY! [00007720]; isotypes: F! [0059182F], G! [00364887], K! [000502897], RB! [00540228], S! [S-R-9700], US! [00004623], VEN! [43808]).

#### 1.2. Dioclea apurensis

Taxon classificationPlantaeFabalesFabaceae

Kunth, Nov. Gen. Sp. 6: 438–439. 1823 [1824].

8D5A45C9-0615-5258-B5FA-E4A33B48E249

Hymenospron apurense (Kunth) Spreng., Syst. Veg. [Sprengel] 4(2): Cur. Post. 282. 1827.
Cymbosema apurense (Kunth) Pittier, Bol. Soc. Venez. Ci. Nat. 7: 154. 1941.

##### Type.

Venezuela, Crescit ad ripam fluminis Orinoci, ad confluentem Apurem, *Humboldt & Bonpland s.n.* (holotype: P! [00660130]; isotype: B-W! [13395-01 0]).

#### 1.3. Dioclea burkartii

Taxon classificationPlantaeFabalesFabaceae

R.H. Maxwell, Darwiniana 16(1–2): 413–416, f. 1–2. 1970.

E696F0D8-4929-537B-B2EF-6AF93D0200DB

##### Type.

Argentina, Corrientes, Ituzaingo, *Bertoni 5325* (holotype: LIL! [000609]).

#### 1.4. Dioclea fimbriata

Taxon classificationPlantaeFabalesFabaceae

Huber, Bol. Mus. Goeldi Hist. Nat. Ethnogr. 5(2): 409–410. 1909.

31A90200-0770-5A71-9929-399A62C5EF67

##### Type.

Brazil, Pará, Prainha, rio Marapy, *Ducke 3577* (lectotype, designated here amongst the syntypes: MG! [003577], photo and fragments F! [0059185F]).

#### 1.5. Dioclea guianensis

Taxon classificationPlantaeFabalesFabaceae

Benth., Comm. Legum. Gen.: 70. 1837.

B78578AA-D496-5891-8B2D-9C99CA660CBA

Dioclea guianensis var. villosior Benth., J. Bot. (Hooker) 2(10): 60. 1840. Type: Guyana, *Schomburgk 629* (lectotype, designated here amongst the isotypes: K! [000502839]; isolectotypes BM! [000931784], BR! [0000005170203], G! [00364900], LE! [00002536], NY! [00007726], P! [02961764], US! [00004616]).
Dioclea panamensis Duchass. ex Walp., Flora 36: 229. 1853. Type: Panama, *Duchassaing s.n.* (holotype: GOET! [004985]).
Dioclea comosa var. panamensis (Duchass. ex Walp.) Kuntze, Revis. Gen. Pl. 1: 179. 1891. Type: based on Dioclea panamensis Duchass. ex Walp.

##### Type.

Guyana, *Schomburgk 83* (lectotype, designated here amongst the isotypes: K! [000502841]; isolectotypes: BM! [000931784], E! [00531193], F! [0059187F], GH! [00277378], K! [000502840], P! [00708474], TCD! [0004427], U! [0003526], US! [00004617]).

#### 1.6. Dioclea holtiana

Taxon classificationPlantaeFabalesFabaceae

Pittier ex R.H. Maxwell, Ann. Missouri Bot. Gard. 77(3): 584. 1990.

F58D7112-2C02-54D4-A53A-31DADD510E41

##### Type.

Venezuela, Amazonas, Boca del Vichada, *Holt & Gehriger 224* (holotype: US! [00004615]; isotype: VEN).

#### 1.7. Dioclea lasiophylla

Taxon classificationPlantaeFabalesFabaceae

Mart. ex Benth., Comm. Legum. Gen.: 70. 1837.

85C7173E-AF86-5EB4-9DDA-269B055F504F

Dioclea guianensis var. lasiophylla (Mart. ex Benth.) R.H. Maxwell ex G.P. Lewis, Legumes Bahia: 254. 1987.

##### Type.

Brazil, Bahia, Cachoeira, *Martius s.n. Obs. 2040* (lectotype, designated here amongst the isotypes: M! [0240656]; isolectotype: M! [0240657]).

#### 1.8. Dioclea lehmannii

Taxon classificationPlantaeFabalesFabaceae

Diels, Biblioth. Bot. 116: 97. 1937.

E29D9210-67FD-5AC4-A3D5-6057370ABC75

##### Type.

Ecuador, Guayas, Naranjal (Naravjae), *Lehmann 5754* (holotype: B†; lectotype, designated here amongst the isotypes: K! [000502891]; isolectotypes: F, K! [000502892], US).

#### 1.9. Dioclea macrantha

Taxon classificationPlantaeFabalesFabaceae

Huber, Bol. Mus. Goeldi Hist. Nat. Ethnogr. 5: 408. 1909.

47055C3C-FEDE-5879-B98A-48914A430F87

##### Type.

Brazil, Pará, Almeirim, *Ducke 3484* (holotype: MG! [003484]; isotype: G! [00364766]).

#### 1.10. Dioclea ovalis

Taxon classificationPlantaeFabalesFabaceae

R.H. Maxwell, Novon 21(2): 227–229, f. 1. 2011.

9FF79F95-3052-55BA-ADFA-FA8730227ABD

##### Type.

Colombia, Cundinamarca, Pacho, *Uribe 1648* (holotype: US! [01050065]; isotype: COL).

#### 1.11. Dioclea sericea

Taxon classificationPlantaeFabalesFabaceae

Kunth, Nov. Gen. Sp. 6: 437–438, pl. 576. 1823 [1824].

6A362A33-7401-5914-AB06-B4E11DE235BC

Hymenospron sericeum (Kunth) Spreng., Syst. Veg. [Sprengel] 4(2): Cur. Post. 283. 1827.

##### Type.

Colombia, Honda, *Humboldt & Bonpland 1681* (lectotype, designated here amongst the isotypes: P! [00708483]; isolectotype: P! [00708482]).

#### 1.12. Dioclea vallensis

Taxon classificationPlantaeFabalesFabaceae

R.H. Maxwell, Novon 21(2): 229–232, f. 2A–K. 2011.

B4B85DAF-7923-5FF4-8CB8-1BD4E5EACEA4

##### Type.

Colombia, Valle del Cauca, río Cajambre, *Cuatrecasas 17499* (holotype: US! [01050066]; isotype: F).

#### 1.13. Dioclea virgata

Taxon classificationPlantaeFabalesFabaceae

(Rich.) Amshoff, Meded. Bot. Mus. Herb. Rijks Univ. Utrecht 52: 69. 1939.

F2E2CBE1-2B35-55C5-999E-3782E03B607C

Dolichos virgatus Rich., Actes Soc. Hist. Nat. Paris: 1: 111. 1792.
Mucuna virgata Desv. ex Steudel, Nomencl. Bot. (ed. 2) 2(9): 164. 1841.

##### Type.

French Guiana, *Leblond 182* (lectotype, designated here amongst the isotypes: P! [00708485]; isolectotype: G! [00364885]).

##### Note.

The specimen in P provides no information concerning its collector, but that information is recorded on the duplicate at G and agrees with the information of the protologue (Richard 1792).

#### 1. 13. 1. Dioclea virgata (Rich.) Amshoff var. virgata

Taxon classificationPlantaeFabalesFabaceae

794135D6-7249-5755-847C-267558EB5D76

Dioclea lasiocarpa Mart. ex Benth., Comm. Legum. Gen.: 69. 1837. Type: Brazil, Bahia, Salvador (‘Soteropolis’), *Martius s.n. Obs. 2016* (lectotype, designated here amongst the syntypes: M! [0240665]; isolectotypes: M! [0240664], M! [0240663]). Note: Bentham (1837) did not cite any specimen for his species D. lasiocarpa. He recognised three unnamed varieties (α, β and ɣ); we selected the specimen cited for var. ‘α’ as the lectotype of the species.
Crepidotropis brasiliensis Walpers, Linnaea 14: 296. 1840. Type: Brazil, Bahia, Cruz de Casma [probably Salvador], *Luschnath s.n.* (lectotype, designated here amongst the isotypes: HAL! [0120300]; isolectotype: LE). Note: Maxwell (1969) said that duplicates in LE are annotated with different numbers (#206, #781, #2054), but probably from the same gathering.
Canavalia bracteolata Merrill, J. Straits Br. Royal As. Soc. 86: 313. 1922. Type: Malaysia, Sabah, Sandakan, (Borneo), *Ramos 1511* (holotype: PHN; isotypes: A! [00059980], BM! [000958604], GH! [00059979], K! [000898374], L! [0018940], P! [00708471], US! [00004634]).
Canavalia peruviana Piper, Publ. Field Mus. Bot. 4: 94. 1925. Type: Peru, La Merced, *Macbride 5551* (holotype: F! [0043480F]; isotypes: G! [00364938], US! [00004655]).

#### 1.13.2. Dioclea virgata var. crenata

Taxon classificationPlantaeFabalesFabaceae

R.H. Maxwell, Ann. Missouri Bot. Gard. 77(3): 585. 1990.

8E0A996C-9AE5-59E5-907B-E816BB49047D

##### Type.

Brazil, Amapá, rio Calcoene, *Pires & Cavalcante 52528* (holotype: U! [1249084]; isotypes: F! [1615326], HUEFS! [27288], NY! [1239737], SP! [000990], S! [S-R-9713], US! [00324272]).

#### 2. Cymbosema

Taxon classificationPlantaeFabalesFabaceae

Benth., J. Bot. (Hooker) 2: 61. 1840.

5ED4272F-CA7C-5B99-A843-846B8540388D

##### Type.

*Cymbosema roseum* Benth.

##### Description.

Woody twining vines. **Stipules** basifixed, not prolonged beyond their base. **Leaves** pinnately trifoliolate, long, stipellate, leaf rachis 5–20 mm. **Inflorescence** an erect pseudoraceme, nodes multiflorous, sessile, secundiflorous; bracteoles chartaceous. **Flowers** with calyx chartaceous, campanulate, the four lobes of almost the same length, upper lobe entire, triangulate, obtuse, lower lobe ovate and acute; petals membranous, bright red, standard petal spreading, rarely reflexed, ecallose, provided with two basal and reflexed auricles, pubescent towards the apex on the outer surface, wing petals as long as the keel, oblong to obovate, provided with a basal spur at the upper margin, keel petals straight, oblanceolate, margins smooth; androecium diadelphous, the vexillary stamen free, the nine remainder fused but free distally, anthers monomorphic, all 10 stamens fertile; intrastaminal nectary disc entire, collar-shaped; pistil almost straight, ovary mostly 5‒6-ovulate, sessile, style not swollen. **Fruits** shortly oblong, 2.4–2.5× longer than wide, up to 2 cm wide, elastically dehiscent, the thin woody valves explosively twisting to release the seeds, upper margin straight, lacking ribs or wings, style persistent and extending as a downcurved rostrum. **Seeds** small, up to 10 mm long and 6 mm wide, lenticular (slightly biconvex); testa hard (bony), smooth; hilum linear, encircling almost half of the seed’s circumference. (Fig. 2E–F).

**Discussion**. Our results support the recognition of *Cymbosema* as a monospecific genus, as originally proposed by Bentham (1840, 1859) and maintained by Maxwell (1970). Zamora (2000) synonymised *Cymbosema* in *Dioclea*, a proposal that is not supported by our results, which recovered *Cymbosema* as sister to *Cleobulia* rather than to *Dioclea*.

*Cymbosema* is diagnosed as having flowers with a diadelphous androecium with the vexillary stamen free, petals bright red, the standard petal spreading (only rarely reflexed), keel petals with smooth margins and fruits oblong and falcate.

Distributed in the Amazon region, extending north to the Pacific coast of Mexico in wet forests.

#### 2.1. Cymbosema roseum

Taxon classificationPlantaeFabalesFabaceae

Benth., J. Bot. (Hooker) 2: 60–61. 1840.

61388126-B0DE-5D0F-B621-E5D96FCA197A

Dioclea purpurea Poepp., Nov. Gen. Sp. Pl. 3: 59. 1845. Type: Brazil, Amazonas, Tefé, *Poeppig D-2619* (holotype: W! [0048636]).
Dioclea rosea (Benth.) N. Zamora, Novon 10: 179. 2000. Type: based on Cymbosema roseum Benth.

##### Type.

Brazil: Rio Branco (Roraima), *Schomburgk 850* (lectotype, designated by Maxwell 1970: K! [000502745]; isolectotypes: BM! [000931430], F! [V0059084F], K! [000502746], US! [00004551], W! [1889-0020599]).

#### 3. Cleobulia

Taxon classificationPlantaeFabalesFabaceae

Mart. ex Benth., Comm. Legum. Gen.: 67. 1873.

F64063B0-5E07-5F7F-B0EF-B0789C89FF44

##### Type.

*Cleobulia multiflora* Mart.ex Benth. [= *Cleobulia coccinea* (Vell.) L.P. Queiroz]

##### Description.

Woody vines. **Stipules** basifixed, not prolonged beyond their base. **Leaves** pinnately trifoliolate, the rachis reduced, sometimes absent, stipellate. **Inflorescence** a pseudoraceme, arcuate, nodes multiflorous, sessile, globose, secundiflorous; bracteoles fleshy. **Flowers** resupinate because of the arching inflorescence; calyx fleshy, cylindrical, the 4 lobes much shorter than the tube, upper lobe truncate to slightly emarginate, lower lobe triangulate and acute; petals firmly chartaceous, pink to purple, standard petal spreading or reflexed, ecallose, provided with two basal and reflexed auricles, pubescent towards the apex on the outer surface, wing petals dwarf, ca. 1/3 of the keel length, sagittate, keel petals upcurved with truncate apices; androecium pseudomonadelphous, staminal tube pubescent at the base, anthers monomorphic, all 10 stamens fertile; intrastaminal nectary disc 10-lobed; pistil straight then upcurved ca. 90° in the middle, ovary 6‒8-ovulate, sessile, style not swollen. **Fruits** linear-oblong, 3‒5× longer than wide, elastically dehiscent, the thin woody valves explosively twisting to release the seeds, upper margin straight to undulate, with thin ribs. **Seeds** small, under 10 mm long and 6 mm wide, lenticular (slightly biconvex); testa hard (bony), smooth; hilum linear encircling almost half of the seed’s circumference (Fig. 2A–D).

**Discussion**. Since first being described, *Cleobulia* was distinguished from *Dioclea* by having dwarf wings with a semi-sagitate blade (Bentham 1837; see Fig. 2B). *Cleobulia* could likewise be diagnosed by having an inflorescence with a long and arching peduncle, leaving its flowers resupinate (i.e. with the standard petal in a lower position and the keel above), a pseudomonadelphous androecium, the base of the staminal tube pubescent, with uniform anthers, a 10-lobed intrastaminal disc, and a sessile and straight ovary.

Three species are found from eastern Brazil to the eastern Brazilian Amazon and one species from western-central Mexico, all mostly in semi-deciduous forests.

#### 3.1. Cleobulia coccinea

Taxon classificationPlantaeFabalesFabaceae

(Vell.) L.P. Queiroz comb. nov.

1A93F318-97E4-5C0B-85D1-589FF4F6959E

urn:lsid:ipni.org:names:77212303-1

Basionym: Dolichos coccineus Vell., Fl. Flumin.: 321, 1829 [1825]. Ic. 7 pl. 158. 1831. Type: Brazil, Rio de Janeiro, “Habitat silvis, fruticetisque maritimis”, Vellozo (lectotype, designated here: plate 158 in *Florae Fluminensis* vol. 7, Vellozo 1831).Epitype: Brazil, Bahia, Lençóis, *L.P. Queiroz et al. 16029* (epitype, designated here: HUEFS! [200008453]; isoepitypes: ALCB! [046364!], RB! [1173635!], US! [3698469]).
Cleobulia multiflora Mart. ex Benth., Comm. Legum. Gen.: 67. 1837. Type: Brazil, Minas Gerais, *Martius s.n.* (lectotype, designated here: M! [0240673]), syn. nov.

##### Note.

A link between *Dolichos coccineus* Vell. and *Cleobulia multiflora* Mart. ex Benth. was established by Maxwell (1977), who speculated that they could be synonymous. The description provided by Vellozo (1829: 321) is exceedingly brief, but presents some traits characteristic of this species, such as flowers small and perianth purpureum. The illustration provides more elements to confirm its identity as *C. multiflora* as it shows resupinate flowers with the standard spreading, the wing petals sagittate and much shorter than the others and the pistil with a straight ovary and style upcurved ca. 90°. There are issues regarding the publication dates of several sections of the Florae Fluminensis but the main text in volume 1 (pages 1 to 329) is considered as having been distributed between 7 September to 28 November 1829 and the illustration volumes on 29 October 1831 (Carauta 1969, 1972; Stafleu and Cowan 1985; Lima 1995), thus predating and having priority over *Cleobulia multiflora* published by Bentham in 1837. To avoid misinterpretation of the name proposed by Vellozo (1831)), we are designating an epitype with leaf, flowers and fruits.

#### 3.2. Cleobulia crassistyla

Taxon classificationPlantaeFabalesFabaceae

R.H. Maxwell, Phytologia 51: 361. 1982.

35B8C88A-6182-5258-9A54-36D2E2DF6224

##### Type.

Mexico, Guerrero, Galeano, *Hinton 14996* (holotype: RSA! [LAM] [0003239]; isotypes: K! [000297082], LL! [00371269], NY! [00006420], US! [00067941]).

#### 3.3. Cleobulia diocleoides

Taxon classificationPlantaeFabalesFabaceae

Benth., Fl. Bras. 15(1): 168. 1859.

27854F8D-66A2-5A03-AC09-C2039C47C668

##### Type.

Brazil, Minas Gerais, *Saint Hilaire s.n. Cat. 1311* (holotype: P! [00758522]). Epitype (designated here): Brazil, Bahia, Campo Formoso, *Queiroz et al. 16306* (HUEFS! [000274630]).

##### Note.

The holotype is the only remanant of the material used by Bentham (1859) for describing *C. diocleoides*. The material now consists of a branch with leaves and a dissected flower bud within an envelope. A detached calyx from a mature flower is the only element that allows us to check that this plant presents flowers much larger than the other species of *Cleobulia* as described by Bentham (1859) and Maxwell (1977). We selected an epitype from a more complete material with flowers and immature fruits.

#### 3.4. Cleobulia leiantha

Taxon classificationPlantaeFabalesFabaceae

Benth., Fl. Bras. 15(1): 162. 1859.

1BAA07A3-2F44-5D9B-A871-E75C107A5922

Cleobulia multiflora var. leiantha (Benth.) R.H. Maxwell, Phytologia 38: 57. 1977.

##### Type.

Brazil, Pará, Santarém, *Spruce [10 03*] (lectotype, designated here from the syntypes: K! [000502886]; isolectotypes: FI! [009795], G! [00364892], K! [000930235], M! [0240670], NY! [00006421], P! [00708488], TCD! [0004431]).

##### Note.

When describing the new species *C. leiantha*, Bentham (1859) cited the specimen collected by Spruce near Santarém. We selected as the lectotype the specimen with a handwritten label and with the collection number 1003 found in other duplicates.

#### 4. Macropsychanthus

Taxon classificationPlantaeFabalesFabaceae

Harms in K. Schumann & Lauterbach, Fl. Schutzgeb. Südsee 366. 1900.

2763AD02-7C5E-53AF-AB8A-E2A96BE5FADA

##### Type.

*Macropsychanthus lauterbachii* Harms.

##### Description.

**Figure 3.**
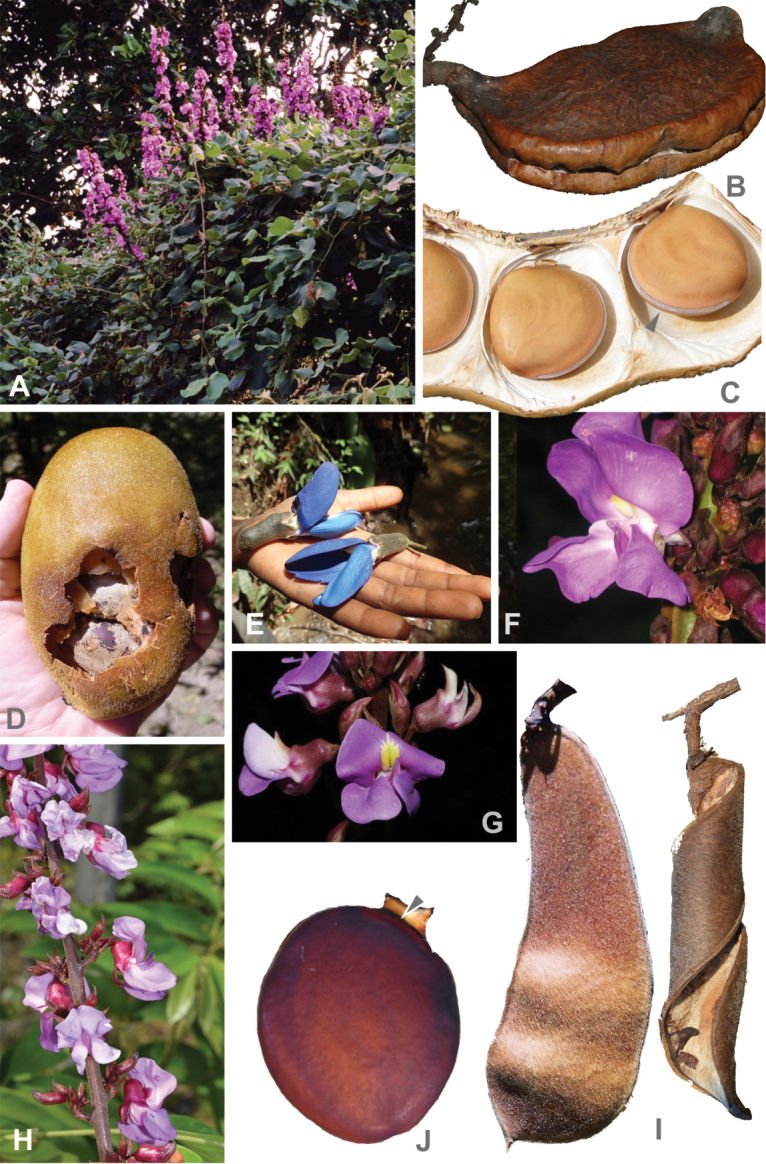
Representatives of the clade B. Macropsychanthus subg. Macropsychanthus (**A–F**). *Macropsychanthus grandiflorus* (Mart. ex Benth.) L.P. Queiroz & Snak **A** flowering vine (from *Queiroz 15227*). *Macropsychanthus marginatus* (Benth.) L.P. Queiroz & Snak **B** mature fruit showing dehiscence through the lower suture only **C** one of the valves removed to show the seeds with a long linear hilum (arrow; from *Queiroz 15225*). *Macropsychanthus edule* (Kuhlm.) L.P. Queiroz & Snak **D** the indehiscent and fleshy fruit decahing to release the seeds (from *Popovkin 1546*). Macropsychanthus lauterbachii Harms var. lauterbachii**E** giant flowers with bluish petals (unvouchered). *Macropsychanthus megacarpus* (Rolfe) L.P. Queiroz & Snak **F** flower (from *Queiroz 10135*). Macropsychanthus subg. Platylobium (**G–J**). *Macropsychanthus scabrus* (Rich.) L.P. Queiroz & Snak **G** flowers (from *Cardoso 2907*). *Macropsychanthus bicolor* (Benth.) L.P. Queiroz & Snak **H** part of the pseudoracemous inflorescence **I** mature (left) and dehisced (right) fruits **J** seed, showing the short hilum (arrow; from *Queiroz 15874*). Photos **A–C, F, H–J**: L.P. Queiroz; D: A. Popovkin; **E**: A.D. Poulsen; **G**: D. Cardoso.

Stout, high-climbing lianas with twining stems, less frequently shrubs or woody vines in open habitats. **Stipules** medifixed and prolonged below their insertion (peltate) or basifixed and not prolonged below their insertion. **Leaves** pinnately trifoliolate, stipellate or estipellate. **Inflorescence** a stout, woody, erect pseudoraceme, nodes multiflorous, woody, stalked and secundiflorous; bracteoles fleshy. **Flowers** massive; calyx with the tube fleshy coriaceous, upper edge convex or humped, 4-lobed, with the upper lobe either entire and triangulate to obtuse or emarginate and then with the resulting tips rounded or 5-lobed with the two upper lobes rounded and the other three lobes triangulate, the lower lobe much longer than the remaining lobes or deeply bilabiate with two oblong lips; petals firm, the standard petal reflexed, somewhat fleshy, bicallose, provided with two basal and folded auricles, wing petals ca. twice as long as the keel, obliquely oblong, obliquely ovate, obovate, elliptic to almost quadrate, basal spur at the upper margin present or lacking, keel upcurved, the keel petals triangular or semi-lunar, extending distally into a slender, obtuse or truncate beak; androecium pseudomonadelphous, the 10 stamens joined in a tube, but the filament of the vexillary stamen free at the base, anthers mostly dimorphic, 5 fertile alternating with 5 sterile or 6 fertile and 4 sterile or anthers uniform and all 10 fertile; intrastaminal nectary disc 10-dentate or 10-lobed; ovary sessile, style usually swollen distally. **Fruit** indehiscent, passively dehiscent or elastically dehiscent with twisting woody valves, turgid, slightly compressed or flat compressed, valves coriaceous, fleshy or woody, upper margin smooth or provided with ribs or wings. **Seeds** 3–5 to 9, massive, either orbiculate and slightly compressed with a hard testa or soft overgrown and without a definite shape, with flat contact planes or elliptic and flat compressed; hilum linear, encircling 1/2 to 4/5 of the seed’s circumference or short and oblong. Fig. 3.

**Discussion**. *Macropsychanthus* Harms is the earliest validly-published genus name for this group. Two older names, *Lepidamphora* Zolling. and *Taurophtalmum* Duchaiss., were not validly published. *Lepidamphora volubilis* Zolling. was published as a synonym of *Dioclea javanica* Benth. with the citation of two specimens (“*Herb. n. 763 et 867 Z.*”; Miquel 1855: 217). *Lepidamphora volubilis* was probably just a name on herbarium sheets and is invalid because it was published as a synonym (ICN Article 36.1; Turland et al. 2018) and because it was published as a species, but the genus to which it was assigned was not validly published at the same time or was not validly published previously (Art. 35.1; Turland et al. 2018).

**Figure 4.**
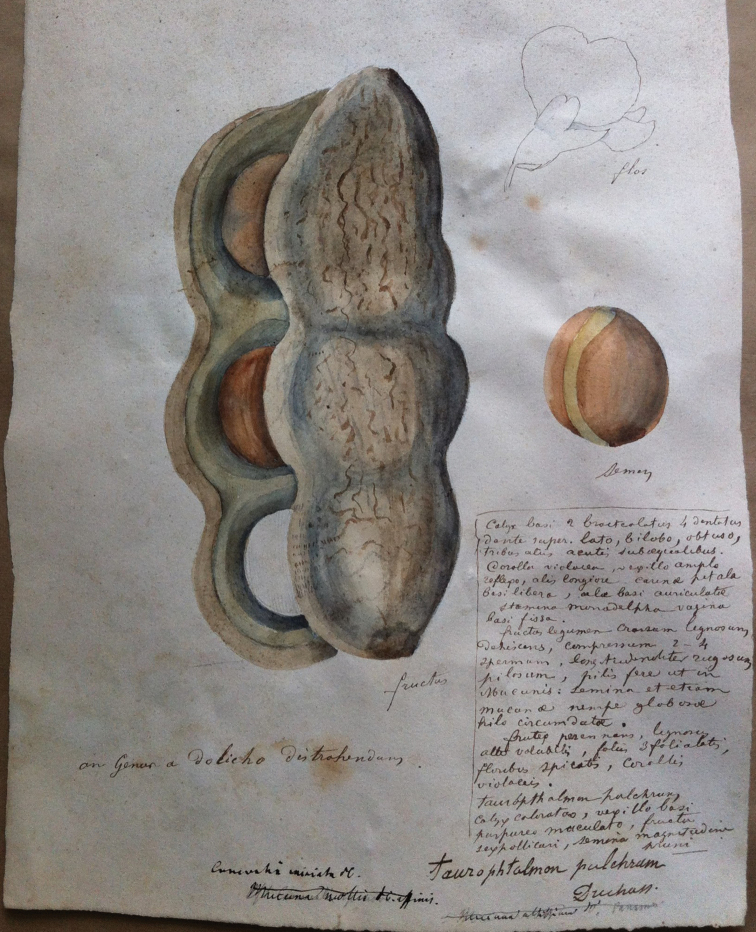
Lectotype of *Taurophtalmum pulchrum* Duchaiss. This watercolour painting housed at GOET is the only remnant of the original material of this species cited in Griesebach (1866).

The Panamanian *Taurophtalmum pulchrum* Duchaiss. was another invalidly-published name that could be related with *Macropsychanthus* as defined here. It was originally published as a synonym of *Canavalia miniata* (Kunth) DC. by Griesebach (1866: 76). However, Urban (1899: 473) placed *T. pulchrum* as a synonym of *Dioclea reflexa* Hook. f. (= *Macropsychanthus comosus*), based on the calyx description provided earlier by Grisebach (1866). The only specimen of *Canavalia* or *Dioclea* collected by Duchassaing that we were able to track is the type of *Dioclea panamensis* Duchaiss. ex Walp. (*Duchassaing s.n.* [GOET 004985]), which is a synonym of *Dioclea guianensis* Benth. and thus does not belong to *Macropsychanthus* as circumscribed here. There is a plate from Duchaissang housed at GOET (and annotated as *Canavalia miniata* by Griesebach) that probably represents the only remnant of the original material of *Taurophtalmum pulchrum*. It is a watercolour painting of a fruit and a seed with a pencil sketch of a flower and a detailed description by Duchaissang (Fig. 4). The fruit represented probably belongs to *Macropsychanthus megacarpus* and not to *M. comosus* as supposed by Urban (1899). The name *Taurophtalmum* literally means “bulls eye” and was probably derived from the Spanish name “ojo de buey” for several species of *Macropsychanthus* (also common in Portuguese as “olho-de-boi”), but not for species of *Dioclea*. In the absence of a specimen and taking the painting in GOET as evidence, we are considering *Taurophtalmum* as related to *Macropsychanthus*, although it is an invalid name.

Two major clades were recovered corresponding to the circumscription of *Macropsychanthus* proposed here. One (clade D) brings together species formerly ascribed to the genera *Luzonia* and *Macropsychanthus*, as well as to Dioclea subg. Pachylobium and *Dioclea huberi* (subg. Platylobium sect. Macrocarpon; Maxwell 2011). Clade C comprises all of the other species formerly ascribed to Dioclea subg. Platylobium. Clade D includes species with mostly medifixed stipules, fruits indehiscent or passively dehiscent and turgid seeds with a long, linear hilum; clade C includes species with basifixed stipules, fruits flat-compressed and elastically dehiscent and seeds with a short and oblong hilum. Our finding that the puzzling *Dioclea huberi* (formerly classified in subg. Platylobium sect. Macrocarpon) is part of clade D blurs the distinction between those major clades, because it shares basifixed stipules and flat-compressed fruits and seeds with D. subg. Platylobium, but seeds with a long linear hilum with D. subg. Pachylobium. Likewise, *Dioclea macrocarpa*, recovered in clade C, shows the basifixed stipules and the short and oblong hilum of D. subg. Platylobium together with the turgid fruits and seeds of D. subg. Pachylobium. Thus, clades B and C are diagnosed by only a few morphological traits (see below) and we chose to recognise them as subgenera of a largely polymorphic genus instead of treating them as two separate genera.

*Macropsychanthus* is a pantropical genus with 46 species. It is most diverse in the New World (36 species), with eleven species from the Philippines and Indonesia to New Guinea and two Pantropical sea-drifted species extending to continental Africa and Madagascar.

#### 4.1. Macropsychanthus Harms subg. Macropsychanthus

Taxon classificationPlantaeFabalesFabaceae

E5B4C97B-78A4-585D-A18D-7A18B49F8880

Dioclea sect. Pachylobium Benth., Comm. Legum. Gen.: 69. 1837. Lectotype [designated here]: Dioclea violacea Mart. ex Benth.
Lepidamphora Zoll., Fl. Ned. Ind. 1(1): 217. 1855. Type: Lepidamphora volubilis Zoll. [= Macropsychanthus comosus (G. Mey.) L.P. Queiroz & Snak], nom. inval. pro syn.
Taurophtalmum Duchass. in Griesebach, Cat. Pl. Cub.: 76. 1886. Type: Taurophtalmum pulchrum Duchaiss. [= Macropsychanthus megacarpus (Rolfe) L.P. Queiroz & Snak], nom. inval. pro syn.
Luzonia Elmer, Leafl. Philipp. Bot. 1: 220. 1907. Type: Luzonia purpurea Elmer.
Dioclea subg. Pachylobium (Benth.) R.H. Maxwell, Novon 21(2): 234. 2011. Type: based on Dioclea sect. Pachylobium Benth.

##### Description.

Stipules medifixed, prolonged below their insertion. Leaves stipellate, stipels mostly setaceous. Fruit indehiscent or passively dehiscent, turgid, slightly compressed (elastically dehiscent with twisting woody valves only in *M. huberi*). Seeds with a long and linear hilum encircling 1/2 to 4/5 of the seed’s circumference (Fig. 3A–F).

The distribution of this section is the same as that of the genus. Species of subg. Macropsychanthus are typical rainforest elements, where they occur as high-climbing lianas over the tallest trees. Few species are found in the savannahs of central Brazil or in the seasonally-dry woodlands of South America.

#### 4.1.1. Macropsychanthus apiculatus

Taxon classificationPlantaeFabalesFabaceae

(R.H. Maxwell) L.P. Queiroz & Snak comb. nov.

1D0429E8-CF82-5A16-9764-DDEA60E530E6

urn:lsid:ipni.org:names:77212304-1

Basionym: Dioclea apiculata R.H. Maxwell, Novon 21(2): 235-237. 2011. Type: Bolivia, La Paz, N Yungas, near Coroico, *Buchtien 664* (holotype: MO; isotypes: F! [588818], G! [00364742]).

#### 4.1.2. Macropsychanthus aureus

Taxon classificationPlantaeFabalesFabaceae

(R.H. Maxwell) L.P. Queiroz & Snak comb. nov.

867DB057-7496-57F0-BA08-490659A76933

urn:lsid:ipni.org:names:77212305-1

Basionym: Dioclea aurea R.H. Maxwell, Ann. Missouri Bot. Gard. 67(3): 664–665. 1981. Type: Colombia, Caldas, Pueblo Rico, *Sneidern 5555* (holotype: S! [S-R-9703]; isotype: NY! [01365123]).

#### 4.1.3. Macropsychanthus carolinensis

Taxon classificationPlantaeFabalesFabaceae

Kanehira & Hosokawa, Trans. Nat. Hist. Soc. Taiwan 24: 414. 1934.

9F4E1307-E180-5A9F-93E2-E62BA2D1C336

##### Type.

Caroline Islands, Palau, *Kanehira 1711* (holotype: TAI!; isotype: P! [02752991]).

#### 4.1.4. Macropsychanthus circinatus

Taxon classificationPlantaeFabalesFabaceae

(R.H. Maxwell) L.P. Queiroz & Snak comb. nov.

3BD12F23-1C4E-5A86-855F-31408828E1E9

urn:lsid:ipni.org:names:77212306-1

Basionym: Dioclea circinata R.H. Maxwell, Novon 21(2): 237. 2011. Type: Colombia, Meta, *Phillipson et al. 1405* (holotype: COL! [000001743]; isotypes: BM! [000931783], MEDEL! [000156], S! [S-R-9704], US! [01050064]).

#### 4.1.5. Macropsychanthus comosus

Taxon classificationPlantaeFabalesFabaceae

(G. Mey.) L.P. Queiroz & Snak comb. nov.

81AB5A7A-8B28-5FB5-9C6D-26A6C5AD367E

urn:lsid:ipni.org:names:77212307-1

Basionym: Dolichos comosus G. Mey, Prim. Fl. Esseq. 241. 1818. Type: Guyana, Essequibo, *Rodschied 93* (holotype: GOET! [004986]).
Dioclea reflexa Hook. f., Niger Fl. 306–307. 1849. Type: West Africa: Cape Palmas and region of Fernando Poo, *Vogel 32* (holotype: K; isotype: GH! [00066325]), syn. nov.
Lepidamphora volubilis Zoll., Fl. Ned. Ind. 1(1): 217. 1855, nom. inval. pro syn. Type: Guyana, Essequibo, *Rodschied 93* (holotype: GOET! [004986]).
Dioclea comosa (G.Mey.) Kuntze, Revis. Gen. Pl. 1: 179. 1891. Type: based on Dolichos comosus G. Mey.

#### 4.1.6. Macropsychanthus densiflorus

Taxon classificationPlantaeFabalesFabaceae

(Huber) L.P. Queiroz & Snak comb. nov.

B1B7724F-71CB-5851-8DAC-F1654D266620

urn:lsid:ipni.org:names:77212308-1

Basionym: Dioclea densiflora Huber, Bol. Mus. Goeldi Hist. Nat. Ethnogr. 5(2): 406–407. 1909. Type: Brazil, Pará, Oriximiná, *Ducke s.n. MG 7903* (holotype: MG! [007903]; isotype: RB! [00174878]).

##### Note.

Huber (1909: 406–407) did not cite any specimen in the original description of *Dioclea densiflora* and, in the absence of a type, Maxwell (1969: 254–255) indicated the specimen *Ducke s.n. RB 11744* (collected on 20 Dec 1919) as a neotype. However, in the introductory pages of his work, Huber (1909) stated that all species were described, based on specimens collected by A. Ducke from 1902 to 1907 and housed at the Museu Goeldi herbarium (MG). He also transcribed Ducke’s field notes showing that he collected in Oriximiná in December of 1906 (Huber 1909: 301), which coincides with the date and locality of the specimen *A. Ducke s.n. MG 7903*. Thus, we are assuming that this specimen is the same one used by Huber (1909) when describing the new species and consider the material housed at MG as the holotype.

#### 4.1.7. Macropsychanthus dictyoneurus

Taxon classificationPlantaeFabalesFabaceae

(Diels) L.P. Queiroz & Snak comb. nov.

5584022B-E424-5013-8317-7BC482CC6196

urn:lsid:ipni.org:names:77212309-1

Basionym: Dioclea dictyoneura Diels, Biblioth. Bot. 116: 97. 1937. Type: Colombia, Putumayo, La Concepción, *Cuatrecasas 10836* (neotype, here designated: COL! [000054481]).

##### Note.

The holotype of *Dioclea dictyoneura* (*Diels 929*) came from Puyo, in Napo-Pastaza, in Ecuadorian Amazon. It was housed at B and was destroyed and we could not trace any duplicate. Maxwell (1969) cited four other specimens, from which we choose as the neotype the material from Concepción as it fits the protologue and was encountered ca. 280 km distant from the area where the original type was collected in the southern Colombian Amazon.

#### 4.1.8. Macropsychanthus dolichobotrys

Taxon classificationPlantaeFabalesFabaceae

Holth., Blumea 5: 192. 1942.

963CD259-522E-5DF3-ABB0-95F8C6623CCF

##### Type.

Indonesia, Talaud Islands, Pasir Malap, *Lam 3002* (holotype: L! [0019084]; isotypes: BO, L! [0019085], L! [0019086]).

#### 4.1.9. Macropsychanthus edulis

Taxon classificationPlantaeFabalesFabaceae

(Kuhlm.) L.P. Queiroz & Snak comb. nov.

8D78AC71-8EEE-5B80-ACCA-6A441487D45B

urn:lsid:ipni.org:names:77212310-1

Basionym: Dioclea edulis Kuhlm., Anais Reunião Sul-Amer. Bot. 3: 79, pl. 6–7. 1940. Type: Espírito Santo, Linhares, Picada da Lagoa do Braz, *Kuhlmann 218* (holotype: RB! [00540230] + fruit coll. RB! carpo [00770250]; isotypes: RB! [00755077], RB! [00755078]).

#### 4.1.10. Macropsychanthus ferrugineus

Taxon classificationPlantaeFabalesFabaceae

Merr., Philipp. J. Sc. 5, Bot.: 121. 1910.

9938964A-2E58-5342-9C14-658425B2999D

Dioclea decandra Amshoff ex Adema, Blumea 43: 234. 1998. Type: based on Macropsychanthus ferrugineus Merr.

##### Type.

Philippines, Mindanao, Lake Lanao, *Clemens 419* (lectotype, designated by Adema 1998: US! [00004643]; isolectotypes: F! [0059545F], K! [000900292], K! [000900293]).

##### Note.

The transfer of *M. ferrugineus* to *Dioclea* was proposed by Amshoff in an unpublished manuscript and validated by Adema (1998). As the name *Dioclea ferruginea* was already occupied by *D. ferruginea* Ducke, Adema (1998) proposed the new name *Dioclea decandra*. However, the original name *M. ferrugineus* is its correct name in *Macropsychanthus* [see also note under *M. duckei*].

#### 4.1.11. Macropsychanthus flexuosus

Taxon classificationPlantaeFabalesFabaceae

(Ducke) L.P. Queiroz & Snak comb. nov.

B7ACCAC6-11D7-5758-8377-FC53C29A1AD4

urn:lsid:ipni.org:names:77212311-1

Basionym: Dioclea flexuosa Ducke, Arch. Jard. Bot. Rio de Janeiro 4: 92–93. 1925. Type: Brazil, Pará, Rio Branco de Óbidos, *Ducke s.n. RB 17271* (holotype: RB! [00616992]; isotypes: RB! [00540232], RB! [00616991]).

#### 4.1.12. Macropsychanthus funalis

Taxon classificationPlantaeFabalesFabaceae

(Poepp.) L.P. Queiroz & Snak comb. nov.

CBD1B206-0737-553F-B094-BFFE9B9AEF79

urn:lsid:ipni.org:names:77212312-1

Basionym: Dioclea funalis Poepp., Nov. Gen. Sp. Pl. 3: 59. 1845. Type: Peru, Pampagaio, *Poeppig 1452* (holotype: W! [0048638]; isotypes: F! [0043445F], NY! [00007725], W! [0048637]).

#### 4.1.13. Macropsychanthus glabrus

Taxon classificationPlantaeFabalesFabaceae

(Benth.) L.P. Queiroz & Snak comb. nov.

4014431E-A075-54AC-A685-EC7A19A232E4

urn:lsid:ipni.org:names:77212313-1

Basionym: Dioclea glabra Benth., Comm. Legum. Gen.: 69. 1837. Type: Brazil, Goiás, San Izidro, *Pohl 1578* (lectotype, designated by Maxwell 1990: W! [2002-0002133]; isolectotypes: [as *Pohl s.n.*] K! [000502843], W! [2002-0002132]).
Dioclea leiophylla Ducke, Arch. Jard. Bot. Rio de Janeiro 4: 91–92, pl. 5, 1925. Type: Brazil, Pará, rio Tapajós, *Ducke s.n. RB 17269* (lectotype, designated here from the syntypes: [in two sheets] RB! [00540234] & [00547582]).

#### 4.1.14. Macropsychanthus grandiflorus

Taxon classificationPlantaeFabalesFabaceae

(Mart. ex Benth.) L.P. Queiroz & Snak comb. nov.

5FC663CC-C4B3-5CB1-9FD5-B0D22E21EC3D

urn:lsid:ipni.org:names:77212314-1

Basionym: Dioclea grandiflora Mart. ex Benth., Comm. Legum. Gen.: 68–69. 1837. Type: Brazil, Bahia, Juazeiro, *Martius 2406* (holotype: M! [0240655]).

#### 4.1.15. Macropsychanthus grandistipulus

Taxon classificationPlantaeFabalesFabaceae

(L.P. Queiroz) L.P. Queiroz & Snak comb. nov.

64BFD12A-6D32-5E2F-A685-CB3388B21D84

urn:lsid:ipni.org:names:77212315-1

Basionym: Dioclea grandistipula L.P. Queiroz, Novon 8(4): 433, f. 1. 1998. Type: Brazil, São Paulo, Iguape, *Cordeiro & Anunciação 1360* (holotype: SP! [000989]; isotypes: HUEFS! [000001844], RB! [00516041]).

#### 4.1.16. Macropsychanthus haughtii

Taxon classificationPlantaeFabalesFabaceae

(R.H. Maxwell) L.P. Queiroz & Snak comb. nov.

011D21E6-5B38-536E-AD0A-66F956614D07

urn:lsid:ipni.org:names:77212316-1

Basionym: Dioclea haughtii R.H. Maxwell, Novon 21(2): 239. 2011. Type: Colombia. Meta, Los Llanos, *Haught 2583* (holotype: COL! [000001747]; isotypes: GH, RB, S! [S-R-9705], US, VEN).

#### 4.1.17. Macropsychanthus hexander

Taxon classificationPlantaeFabalesFabaceae

(Ralph) L.P. Queiroz & Snak comb. nov.

8AD7197F-DE43-57F0-8847-7F4F0919F152

urn:lsid:ipni.org:names:77212317-1

Basionym: Mucuna hexandra Ralph, IC. Carp., 30, t. 34, f. 5. 1849. Type: The plate of Dolichos hexandrus Roxb. (*nom. nud.*), Ic. 2328 (holotype K [available at Kew 2006, http://apps.kew.org/floraindica/displayImages.do?index=6]).
Dolichos coriaceus Graham ex Wall., Numer. List [Wallich] n. 5562. 1831, nom. inval. (nom. nud.). Type: Singapore, Penang, *Wallich Cat. no. 5562* (holotype: K! [001121297]).
Dioclea coriacea (Graham ex Wall.) Rusby, Mem. Torrey Bot. Club 3(3): 22. 1893. Type: based on Dolichos coriaceus Graham ex Wall.
Macropsychanthus novo-guineensis Pulle, Nova Guinea 8: 382. 1910. Type: Indonesia, Irian Jaya, *Versteeg 1028* (lectotype, designated here amongst the syntypes: L! [0018939]; isolectotypes: BO, U! [1248394]).
Dioclea hexandra (Ralph) Mabb., Taxon 29(5–6): 605–606, 1980. Type: based on Mucuna hexandra Ralph.

##### Note.

**Figure 5.**
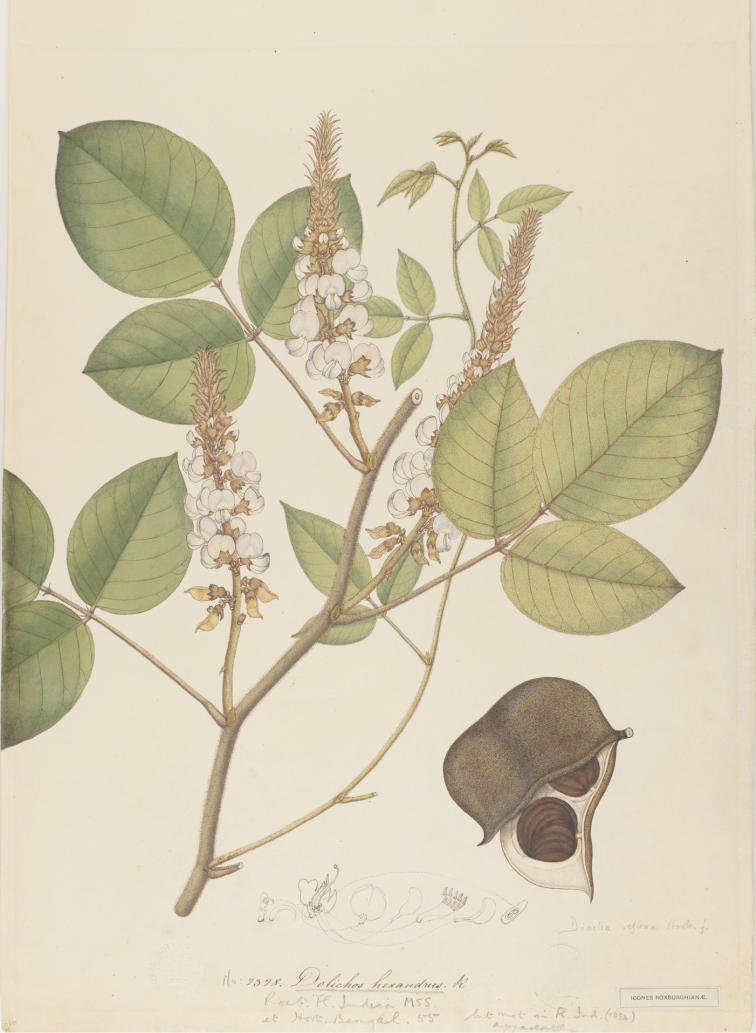
Original painting of Roxburgh icon 2328 (K) from *Dolichos hexandrus* that was used by Ralph (1849) to propose *Mucuna hexandra* Ralph. Note the androecium with six fertile stamens typical of *Macropsychanthus hexander* (Ralph) L.P. Queiroz & Snak. Available Roxburgh‘s Flora Indica (Kew 2006) at http://apps.kew.org/floraindica/displayImages.do?index=6.

Adema (1998) considered that plate 5 of *Parrana rubra* Rumph. in Rumphius (1747) should be taken as the type of *Mucuna hexandra* Ralph. In our opinion, the illustration of *Parrana rubra* does not provide sufficient elements to allow associating it with *Macropsychanthus hexander* (or with any species of *Macropsychanthus*). When publishing *Mucuna hexandra*, Ralph (1849) illustrated the fruit and explicitly stated that he took the drawing from the unpublished painting of *Dolichos hexandrus* in Roxburgh icon 2328 that fits quite well with the diagnostic features of *Mucuna hexandra*, including the androecium with six fertile stamens (Fig. 5). We thus consider the original Roxburgh figure as the holotype of the basionym.

#### 4.1.18. Macropsychanthus huberi

Taxon classificationPlantaeFabalesFabaceae

(Ducke) L.P. Queiroz & Snak comb. nov.

0770EAD6-13D2-530B-9409-4D994BF63D84

urn:lsid:ipni.org:names:77212318-1

Basionym: Dioclea huberi Ducke, Arch. Jard. Bot. Rio de Janeiro 3: 172–173. 1922. Type: Brazil, Pará, Gurupá, *Ducke s.n. MG 16533* (lectotype, designated here amongst the syntypes: [in two parts] RB! [00540233] & [00547679]; isolectotype: S! [S-R-9706]).

#### 4.1.19. Macropsychanthus javanicus

Taxon classificationPlantaeFabalesFabaceae

(Benth.) L.P. Queiroz & Snak comb. nov.

C20B1DAB-DB06-581B-8248-9D7123A1F72F

urn:lsid:ipni.org:names:77212319-1

Basionym: Dioclea javanica Benth., Pl. Jungh. 2: 236. 1852. Type: Indonesia, Java, *Junghuhn s.n. [=108*?] (lectotype, designated here: K! [000898373]; isolectotype: L! [0018938]).
Dioclea fergusonii Thwaites, Enum. Pl. Zeyl. 5: 412. 1864. Type: Sri Lanka, near Colombo, *Ferguson 3817* (holotype: BM! [000958602]; isotypes: G! [00364007], K! [000898372], P! [00708478]).

#### 4.1.20. Macropsychanthus jamesonii

Taxon classificationPlantaeFabalesFabaceae

(R.H. Maxwell) L.P. Queiroz & Snak comb. nov.

B58A1F8B-6A2F-550D-80AA-441DFB0773B7

urn:lsid:ipni.org:names:77212320-1

Basionym: Dioclea jamesonii R.H. Maxwell, Novon 21(2): 239, f. 7. 2011. Type: Ecuador. ‘‘Collectio Reichenbach fil., Acqu. 1889’’, *Jameson s.n.* (holotype: W! [125398]; isotype: W! [125301]).

#### 4.1.21. Macropsychanthus latifolius

Taxon classificationPlantaeFabalesFabaceae

(Benth.) L.P. Queiroz & Snak comb. nov.

E72AAB53-8CDC-52AD-873D-0FF53FA4EFD8

urn:lsid:ipni.org:names:77212321-1

Basionym: Dioclea latifolia Benth., Comm. Legum. Gen.: 69. 1837. Type: Brazil, Goiás?, San Izidro, *Pohl 1565* (lectotype, designated here from the syntypes: W! [2002-0002134]; isotypes: K! [000189688], NY! [00007731]).

#### 4.1.22. Macropsychanthus lauterbachii

Taxon classificationPlantaeFabalesFabaceae

Harms, in Schumann & Lauterb. Fl. Schutzgeb. Südsee 367. 1900.

3889012E-6540-52C7-9275-DB1E4E042590

##### Type.

Papua New Guinea, Nurufluss, *Lauterbach s.n.* (lectotype, designated here from the syntypes: WRSL!; isolectotype: B †).

**4.1.22.1. Macropsychanthus lauterbachii Harms var. lauterbachii in Verdcourt, Kew Bull. 32(2): 455. 1978.**

#### 4.1.22.2. Macropsychanthus lauterbachii

Taxon classificationPlantaeFabalesFabaceae

var. glabricalyx (Verd.) Adema, Blumea 43: 236. 1998.

8758BB21-C34B-5D7E-A0ED-EB8FFD793344

Macropsychanthus lauterbachii subsp. glabricalyx Verd., Kew Bull. 32(2): 456. 1978.

##### Type.

Papua New Guinea, Northern District, near Kokoda, *Hoogland 3953* (holotype: K! [000900297]; isotypes: A! [00057463], BM! [000958600] & [000958601], BRI! [AQ0050313], CANB! [74008.1], L! [0019087], LAE, MEL! [81601], US! [00170444]).

#### 4.1.22.3. Macropsychanthus lauterbachii

Taxon classificationPlantaeFabalesFabaceae

var. hirsutus Verd., Kew Bull. 32(2): 456. 1978.

5011EDF6-87ED-51EE-A66D-A46BC0CD0941

##### Type.

Papua New Guinea, Morobe District: near Lae, *Millar in NGF 13819* (holotype: K! [000900298]; isotypes: A! [00057464], E! [00531192], BRI! [AQ0050930], L! [0019088], LAE).

#### 4.1.22.4. Macropsychanthus lauterbachii

Taxon classificationPlantaeFabalesFabaceae

var. parviflorus (Verd.) Adema, Blumea 43: 236. 1998.

59E7BCBB-CA38-5FE6-94CD-0A476740638E

Macropsychanthus lauterbachii subsp. parviflorus Verd., Kew Bull. 32(2): 456-457. 1978. Type: based on Macropsychanthus lauterbachii var. parviflorus (Verd.) Adema.
Macropsychanthus lauterbachii subsp. neobritannicus Verd., Kew Bull. 32(2): 456-457. 1978. Type: Papua New Guinea, New Britain, Talasea subdistrict, Kopiura river, *Henty in NGF 29391* (holotype: LAE; isotypes: A! [00057465], BOG, BRI! [AQ0052463], CANB, K! [000900299], L! [0019091], SING).

##### Type.

Papua New Guinea, Milne Bay District, Rossel Island, *Brass 28335* (holotype: K! [000900300]; isotypes: A! [00057466], L! [0019089] & [0019090], LAE, S! [S10-10521], US! [00170445]).

#### 4.1.23. Macropsychanthus malacocarpus

Taxon classificationPlantaeFabalesFabaceae

(Ducke) L.P. Queiroz & Snak comb. nov.

DC89C3F3-7E59-59E5-80E4-F4C14A8FBD33

urn:lsid:ipni.org:names:77212322-1

Basionym: Dioclea malacocarpa Ducke, Arch. Jard. Bot. Rio de Janeiro 3: 170–172. 1922. Type: Brazil, Pará, Belém, *Ducke in MG 15808* (lectotype, designated here from the syntypes: MG! [015700]; isolectotypes: BM! [000931774], G! [00364764], RB!, US! [00004611]).

#### 4.1.24. Macropsychanthus marginatus

Taxon classificationPlantaeFabalesFabaceae

(Benth.) L.P. Queiroz & Snak comb. nov.

E1D69DD0-8F3A-5AA2-A412-8D37FA9E255D

urn:lsid:ipni.org:names:77212323-1

> Basionym: Dioclea marginata Benth., Fl. Bras. 15(1): 166. 1859. Type: Brazil, Bahia, near villa da Barra, *Blanchet 3085* (lectotype, designated here from the isotypes: K! [000206534]!; isolectotypes: BM! [000931779], G! [00364023], K! [000206533], LE! [00002537], MO! [2071255], NY! [00007732], P! [00708476]).

#### 4.1.25. Macropsychanthus megacarpus

Taxon classificationPlantaeFabalesFabaceae

(Rolfe) L.P. Queiroz & Snak comb. nov.

7F34D1C3-7CF6-57CF-A8D6-33B3A4931ECA

urn:lsid:ipni.org:names:77212324-1

Basionym: Dioclea megacarpa Rolfe, Bull. Misc. Inform. Kew 1901: 139. 1901. Type: Trinidad, St.’Ann, *Hart 6406* (lectotype, designated by Amshoff (1939): K! [000502846]).
Dioclea reflexa var. grandiflora Benth., Fl. Bras. 15(1): 162. 1859. Type: Brazil, Piauí, inter Boa Esperança et Sant’Anna das Mercês, *Gardner 2117* (lectotype, designated here from the isotypes: K! [000206505]; isotypes: BM! [000931778], K! [000206506]).
Taurophtalmum pulchrum Duchass. *in* Griesebach, Cat. Pl. Cub.: 76. 1886, nom. inval. pro syn. Lectotype [designated here]: watercolour painiting by Duchassaing (GOET!), syn. nov. (Fig. 4).

#### 4.1.26. Macropsychanthus mindanaensis

Taxon classificationPlantaeFabalesFabaceae

Merr., Philipp. J. Sci. 5: 120. 1910.

97A3BBA4-1C62-5924-A61B-2E863745B0E0

##### Type.

Philippines, Mindanao, Province of Surigao, *Bolster 330* (holotype: PNH †).

##### Note.

Merrill (1910) did not refer to the herbarium where the type is housed and we were unable to track it. The PNH herbarium curator confirmed that the holotype was housed at PNH (as PNH 4697) but that it was destroyed during World War II (L. Evangelista, Philippine National Herbarium, National Museum, pers. comm.). Adema (1998) speculated that it could be more closely related to (or conspecific with) *M. ferrugineus* as it was described as having ten fertile stamens.

#### 4.1.27. Macropsychanthus mollicomus

Taxon classificationPlantaeFabalesFabaceae

(Ducke) L.P. Queiroz & Snak comb. nov.

232F85A1-C281-55D9-9DFB-A42A490CE881

urn:lsid:ipni.org:names:77212325-1

Basionym: Dioclea mollicoma Ducke, Trop. Woods 90: 19–20. 1947. Type: Brazil, Amazonas, Esperança, *Ducke 1598* (lectotype, designated here from the syntypes: MG! [018160]; isolectotypes: A! [00277380], F! [0059198F], GH, K! [000978042], NY! [00007734], R! [000054824], RB! [00649170; 00540238], UC! [1204097], US! [00004610]).

#### 4.1.28. Macropsychanthus pulchrus

Taxon classificationPlantaeFabalesFabaceae

(Moldenke) L.P. Queiroz & Snak comb. nov.

E3CD7255-91DE-570E-A297-08A720C7B6CC

urn:lsid:ipni.org:names:77212326-1

Basionym: Dioclea pulchra Moldenke, Phytologia 1(1): 6–7. 1933. Type: Colombia, Boyaca, El Umbo region, *Lawrence 528* (holotype: NY! [00007739]; isotypes: A! [00277304], BM! [000931782], F! [0059201F], FI! [005117], G! [00364763], K! [000502890], MG, MO! [277051], NY! [00007738], S! [S-R-9708], U! [0008110], UC, US! [00004604]).

#### 4.1.29. Macropsychanthus purpureus

Taxon classificationPlantaeFabalesFabaceae

(Elmer) L.P. Queiroz & Snak comb.nov.

7E0BF8CF-0301-55BD-87FB-D0F4089638DC

urn:lsid:ipni.org:names:77212327-1

Basionym: Luzonia purpurea Elmer, Leafl. Philipp. Bot. 1: 220. 1907. Type: Philippines, Luzon, Province of Tayabas, Lucban, May 1907, *Elmer 9013* (holotype: PNH; isotypes: A! [00057462], E! [00301634], L! [0019058], MO! [256507], NY! [00016167], US! [00004668]).

#### 4.1.30. Macropsychanthus rufescens

Taxon classificationPlantaeFabalesFabaceae

(Benth.) L.P. Queiroz & Snak comb. nov.

A1662B47-3133-5473-868E-3BB71FDFD07D

urn:lsid:ipni.org:names:77212328-1

Basionym: Dioclea rufescens Benth., Comm. Legum. Gen.: 69. 1837. Type: Brazil, Minas Gerais?, “Frigna do Alfonso”, *Pohl s.n.* (lectotype, designated here from the isotypes: K! [000189690] [labelled as number 1102]; isolectotypes: F! [0059204F], K! [000189689], NY! [00007743], W! [2002-0002137; 2002-0002138]).
Dioclea rubiginosa Tul., Arch. Mus. Hist. Nat. 4: 72. 1844. Type: Brazil, Minas Gerais, *Claussen 958*, 1838 (lectotype designated here: P! [00708479]; isolectotype: P! [00708480]).

#### 4.1.31. Macropsychanthus schimpffii

Taxon classificationPlantaeFabalesFabaceae

(Diels) L.P. Queiroz & Snak comb. nov.

A40A54C2-F5E0-5AD1-9CA5-C3DD1B3FAC59

urn:lsid:ipni.org:names:77212329-1

Basionym: Dioclea schimpffii Diels, Biblioth. Bot. 116: 97. 1937. Type: Ecuador, Chimborazo, Naranjapata, rio Chanchan, *Schimpff 565* (holotype: B†; lectotype, designated here: G! [00364005]; isolectotypes: MO! [289358; 289359]).

#### 4.1.32. Macropsychanthus schottii

Taxon classificationPlantaeFabalesFabaceae

(Benth.) L.P. Queiroz & Snak, comb. nov.

12CF0FF2-628F-5A48-A1E0-E3A0F8B787B2

urn:lsid:ipni.org:names:77212330-1

Basionym: Dioclea schottii Benth., Comm. Legum. Gen.: 70. 1837. Type: Brazil, Rio de Janeiro, “in campis”, *Schott s.n.* (lectotype, designated here from the isotypes: W! [2002-0002135]; isolectotypes: F! [0059206F], K! [000502844], NY! [00007745], W! [2002-0002136]).

#### 4.1.33. Macropsychanthus sclerocarpus

Taxon classificationPlantaeFabalesFabaceae

(Ducke) L.P. Queiroz & Snak comb. nov.

EDEA513E-C3A0-5587-B486-F0F2DB1D98A5

urn:lsid:ipni.org:names:77212331-1

Basionym: Dioclea sclerocarpa Ducke, Arch. Jard. Bot. Rio de Janeiro 3: 169–170. 1922. Type: Brazil, Pará, Monte Alegre, *Ducke s.n. MG 17152* (lectotype, designated here from the syntypes: RB! [00540242]; isolectotypes: BM! [000931772], MG, P! [02752764]).
Dioclea reflexa var. glabrescens Benth., Fl. Bras. 15(1): 162-163. 1859. Type: Brazil, Maranhão, *Gardner 5988* (lectotype, designated here from the syntypes: K! [000502898]; isolectotypes: BM! [000931773]).

#### 4.1.34. Macropsychanthus ucayalinus

Taxon classificationPlantaeFabalesFabaceae

(Harms) L.P. Queiroz & Snak comb. nov.

37F13019-283C-5495-ACCC-E7852A49F4DF

urn:lsid:ipni.org:names:77212332-1

Basionym: Dioclea ucayalina Harms, Notizbl. Bot. Gart. Berlin-Dahlem 9: 262. 1925. Type: Peru, middle Ucayali, Yarina Cocha, *Tessmann 3464* (holotype: B† [photo F! [F0BN002411]; lectotype, designated here from the isotypes: S! [S-R-9711]; isolectotypes: G! [00364004], NY! [00007748], US! [00004646]).

#### 4.1.35. Macropsychanthus umbrinus

Taxon classificationPlantaeFabalesFabaceae

(Elmer) L.P. Queiroz & Snak comb. nov.

0B20DA49-6941-5832-8EC2-2B55F4BED48A

urn:lsid:ipni.org:names:77212333-1

Basionym: Dioclea umbrina Elmer, Leafl. Philipp. Bot. 1: 224. 1907. Type: Philippines, Leyte, *Elmer 7249* (holotype: PHN; isotype: K! [000898375]).

##### Note.

In the protologue of the basionym, Elmer (1907) cited the type specimen as “9015, A. D. E. Elmer, Palo, Province of Leyte, Leyte, January, 1906”. All of that information is on the label of the Kew specimen, although that label gives the collector number as 7249. As all of the other elements fit the protologue, we are considering the Kew specimen as an isotype.

#### 4.1.36. Macropsychanthus violaceus

Taxon classificationPlantaeFabalesFabaceae

(Mart. ex Benth.) L.P. Queiroz & Snak comb. nov.

BDC57C57-8E05-5A32-BEA6-7717518201F7

urn:lsid:ipni.org:names:77212334-1

Basionym: Dioclea violacea Mart. ex Benth., Comm. Legum. Gen.: 69. 1837. Type: Brazil, Bahia?, Mucuri fluv., *Wied s.n.* (lectotype, designated here from the syntypes: BR! [0000005194667]; isolectotypes: BR [0000005196715; [0000005194995]).
Dolichos altissimus Vell., Fl. Flumin.: 320. 1825 [1829], non Dolichos altissimus Jacq., Enum. Syst. Pl. 27. 1760, nom. illeg. Type: Brazil, Rio de Janeiro, “Habitat silvis maritimis”, Vellozo (lectotype, designated here: tab. 154 in Vellozo, *Fl. Flumin. Ic.* vol. 7, 1829).
Dioclea pilifera Tul., Arch. Mus. Hist. Nat. 4: 71. 1844. Type: Brazil, *Claussen s.n.* (holotype: P! [00708484]).
Dioclea paraguariensis Hassl., Repert. Spec. Nov. Regni Veg. 16: 228–229. 1919. Type: Paraguay, Lake Ypacaray, *Hassler 12460* (lectotype, designated here from the syntypes: G! [00381578]; isolectotypes: C! [10012111], E! [00531190], G! [00381577], K! [000502900], S! [S-R-9701]).
Dioclea altissima (Vell.) Rock, Legum. Pl. Hawaii: 201. 1920. Type: based on Dolichos altissimus Vell.

#### 4.1.37. Macropsychanthus wilsonii

Taxon classificationPlantaeFabalesFabaceae

(Standl.) L.P. Queiroz & Snak comb. nov.

1DAFF7D1-1546-5A76-BEF0-53767F3824A7

urn:lsid:ipni.org:names:77212335-1

Basionym: Dioclea wilsonii Standl., Publ. Field Mus. Nat. Hist., Bot. Ser. 4(8): 310–311. 1929. Type: Honduras, *Wilson 336* (holotype: F! [0059180F]; isotypes: NY! [00007718], US [00004644]).
Dioclea atropurpurea Pittier, Bol. Tecn. Minist. Agric. 5: 79, f. 34, 1944. Type: Venezuela, Sucre, entre Cumaná y Cumanacoa, *Pittier 14660* (holotype: VEN [4439]; isotypes: K! [000502895], S! [S-R-9702]).

#### 4.2. Macropsychanthus

Taxon classificationPlantaeFabalesFabaceae

subg. Platylobium (Benth.) L.P. Queiroz

5137A6FB-CA82-5B1D-84EC-057831869FA2

Dioclea sect. Platylobium Benth., Fl. Bras. 15(1): 164. 1859.
Dioclea sect. Macrocarpon Amshoff, Meded. Bot. Mus. Herb. Rijks Univ. Utrecht 52: 68. 1939. Type [designated by Maxwell, 2011]: Dioclea macrocarpa Huber.
Dioclea subg. Platylobium (Benth.) R.H. Maxwell, Novon 21(2): 232, 2011. Type: based on Dioclea sect. Platylobium Benth.

##### Type.

[designated by Maxwell, 2011]: *Dioclea bicolor* Benth. Stipules basifixed, not prolonged below their insertion. Leaves estipellate. Fruit flat, compressed and elastically dehiscent, with twisting woody valves, rarely indehiscent or passively dehiscent and turgid (*M. ruddiae*). Seeds with a short and oblong hilum (Fig. 3 G–J).

This subgenus fits the circumscription of Dioclea subg. Platylobium (sensu Maxwell, 2011) with the transfer of *Macropsycanthus huberi* to the section Macropsycanthus.

Nine species are known from South America, centred in the Amazon and Guyana region and three species extend southward into the Cerrado biome in central Brazil.

#### 4.2.1. Macropsychanthus bicolor

Taxon classificationPlantaeFabalesFabaceae

(Benth.) L.P. Queiroz & Snak comb. nov.

227E69C9-550F-50CF-8601-146D4BE355AB

urn:lsid:ipni.org:names:77212336-1

Basionym: Dioclea bicolor Benth., Comm. Legum. Gen.: 69. 1837. Type: Brazil, Amazonas [‘Rio Negro’], Coari, *Martius s.n. Obs. 2877* (lectotype, designated here from the syntypes: M! [0240649]; isolectotype: M! [0240648]).
Dioclea rostrata Benth., Comm. Legum. Gen.: 69. 1837. Type: Brazil, “Villa Nova do Almeida”, *Wied s.n.* (lectotype, designated here from the isotypes: BR! [0000005197378]; isolectotype: BR! [0000005197040]), *syn. nov.*
Dioclea rostrata var. nitida Benth., Fl. Bras. 15(1): 168. 1859. Type: Brazil, Mato Grosso?, ‘Salto do Curaú, rio Pardo’, *Riedel 452 (560)* (lectotype, designated here from the isotypes: LE! [00002539]; isolectotypes: A! [00066322], F! [0059202F], K! [000502901], NY! [01583820]), *syn. nov.*

#### 4.2.2. Macropsychanthus coriaceus

Taxon classificationPlantaeFabalesFabaceae

(Benth.) L.P. Queiroz & Snak comb. nov.

AD780ABD-74E2-59F2-9A8F-A253202B7D2C

urn:lsid:ipni.org:names:77212337-1

Basionym: Dioclea coriacea Benth., Comm. Legum. Gen.: 69. 1837. Type: Brazil, Goiás?, Corgo do Padre, *Pohl 1966* (lectotype, designated here from the syntypes: W! [2002-0002131]; isolectotypes: K! [000189687], NY [00007724]).

#### 4.2.3. Macropsychanthus duckei

Taxon classificationPlantaeFabalesFabaceae

L.P. Queiroz & Snak nom. nov.

CEEBEB9C-D6A4-5C88-8AFB-0E1BCA6AB184

Basionym: Dioclea ferruginea Ducke, Arch. Jard. Bot. Rio de Janeiro 4: 93, pl. 7. 1925. Type: Brazil, Pará, rio Tapajós, lago Quataquara, *Ducke in RB 17266* (holotype: RB! in three parts [00616768; 00616767; 00540231]).

##### Note.

The specific epithet of the basionym *Dioclea ferruginea* cannot be used to make a new combination in *Macropsychanthus* because the name *M. ferrugineus* is already occupied. We propose the new name honouring the botanist A. Ducke who made huge contributions to our knowledge of the Amazon flora and discovered this species.

#### 4.2.4. Macropsychanthus erectus

Taxon classificationPlantaeFabalesFabaceae

(Hoehne) L.P. Queiroz & Snak comb. nov.

5D75D21E-6EDA-54B1-8430-8CC5EBC1B4D5

urn:lsid:ipni.org:names:77212338-1

Basionym: Dioclea erecta Hoehne, Comm. Lin. Telegr., Bot. 45(8): 92, t. 151, 159. 1919. Type: Brazil, Mato Grosso, Juruena, *Hoehne 1886* (lectotype, designated here from the syntypes: R! [000211395]).

#### 4.2.5. Macropsychanthus hispidimarginatus

Taxon classificationPlantaeFabalesFabaceae

(R.H. Maxwell) L.P. Queiroz & Snak comb. nov.

F470A554-93A6-5732-A2CF-867BDAADF1DE

urn:lsid:ipni.org:names:77212339-1

Basionym: Dioclea hispidimarginata R.H. Maxwell, Novon 21(2): 232. 2011. Type: Peru, Amazonas, Valle de Rio Santiago, Caterpiza, *Huashikat 1654* (holotype: MO! [713605]; isotype: JEF).

#### 4.2.6. Macropsychanthus macrocarpus

Taxon classificationPlantaeFabalesFabaceae

(Huber) L.P. Queiroz & Snak comb. nov.

E5D8D788-6B82-5C2D-96A9-BDC5924E9F2B

urn:lsid:ipni.org:names:77212340-1

Basionym: Dioclea macrocarpa Huber, Bol. Mus. Goeldi Hist. Nat. Ethnogr. 5(2): 410–411. 1909. Type: Brazil, Pará, rio Ariramba, *Ducke s.n. MG 8071* (holotype: MG! [8071]; isotypes: BM! [000931775], G! [00365046]).

#### 4.2.7. Macropsychanthus rigidus

Taxon classificationPlantaeFabalesFabaceae

(R.S. Cowan) L.P. Queiroz & Snak comb. nov.

1B767239-22CA-5875-9B06-6B16BDD3F699

urn:lsid:ipni.org:names:77212341-1

Basionym: Dioclea rigida R.S. Cowan, Mem. New York Bot. Gard. 10(1): 150–151. 1958. Type: Venezuela: Amazonas, Cerro Paru, *Cowan & Wurdack 31252* (holotype: Y! [00007744]; isotype: US! [00004603]).
Dioclea steyermarkii R.H. Maxwell, Ann. Missouri Bot. Gard. 77(3): 585–587, f. 1. 1990. Type: Venezuela, Amazonas, Atures, *Huber 4476* (holotype: US! [00324271]; isotypes: K! [00324271], MYF, NY! [00007746]), syn. nov.

#### 4.2.8. Macropsychanthus ruddiae

Taxon classificationPlantaeFabalesFabaceae

(R.H. Maxwell) L.P. Queiroz & Snak comb. nov.

9895C53F-04E5-5004-9C9A-97E1B4C25880

urn:lsid:ipni.org:names:77212342-1

Basionym: Dioclea ruddiae R.H. Maxwell, Ann. Missouri Bot. Gard. 75(2): 730–732, f. 1. 1988. Type: Venezuela, Amazonas, Cerro Huachamacari, *Maguire et al. 29930* (holotype: US! [00067942]; isotypes: F! [0059203F], GH! [00066323], K, IAN, MO, NY, P, RB! [00540240], S! [S-R-9709], U! [0003527], VEN! [43782]).

#### 4.2.9. Macropsychanthus scabrus

Taxon classificationPlantaeFabalesFabaceae

(Rich.) L.P. Queiroz & Snak comb. nov.

86ADEBA4-2282-55D2-BB62-E88189B94778

urn:lsid:ipni.org:names:77212343-1

Basionym: Dolichos scaber Rich., Actes Soc. Hist. Nat. Paris 1: 111. 1792. Type: French Guyana, Leblond 183 (holotype: G! [00364886]).
Dioclea scabra (Rich.) R.H. Maxwell, Ann. Missouri Bot. Gard. 77(3): 578. 1990.

##### Note.

Maxwell (1990) designated a neotype for *Dolichos scaber* (*de la Cruz 3090*, UC), but that neotype should be substituted after the finding of the *Leblond* specimen, which was part of a set of plants sent by Leblond from French Guyana (Richard 1792).

#### 4.2.9.1. Macropsychanthus scabrus

Taxon classificationPlantaeFabalesFabaceae

(Rich.) L.P. Queiroz & Snak var. scabrus

80B390BE-8A58-512A-9221-C829C83BEF3F

Dioclea elliptica R.H. Maxwell, Ann. Missouri Bot. Gard. 77(3): 578. 1990, nom. inval. (nom. nud.).

##### Note.

Maxwell (1969) proposed the name *Dioclea elliptica* in his Ph.D. dissertation, using as the type the specimen *de la Cruz 3090* from Essequibo, Guyana. That dissertation is not considered an effective publication, however, under ICN Article 30.9 (Turland et al. 2018). It was later published as a synonym of *D. scabra* by Maxwell (1990), but with no description, thus being a nomen nudum (ICN Art. 38.1, Turland et al. 2018).

#### 4.2.9.2. Macropsychanthus scabrus

Taxon classificationPlantaeFabalesFabaceae

var. brownii (R.H. Maxwell) L.P. Queiroz & Snak comb. nov.

87B3E303-A962-5AAD-80B0-DB88B735AAF2

urn:lsid:ipni.org:names:77212344-1

Basionym: Dioclea scabra var. brownii R.H. Maxwell, Ann. Missouri Bot. Gard. 77(3): 579, 581. 1990. Type: Venezuela, Amazonas, Atabapo, *Davidse et al. 17450* (holotype: MO! [277050]; isotypes: MYF, NY).

#### 4.2.9.3. Macropsychanthus scabrus

Taxon classificationPlantaeFabalesFabaceae

var. schulzii (R.H. Maxwell) L.P. Queiroz & Snak comb. nov.

31CE3431-43E2-56C7-BC44-F3C99F8D0B88

urn:lsid:ipni.org:names:77212345-1

Basionym: Dioclea scabra var. schulzii R.H. Maxwell, Ann. Missouri Bot. Gard. 77(3): 581. 1990. Type: Guyana, Essequibo, Potaro, *Atkinson 116* (holotype: BM! [000931781]; isotypes: NY! [01365181], US).

## Supplementary Material

XML Treatment for Dioclea

XML Treatment for Dioclea albiflora

XML Treatment for Dioclea apurensis

XML Treatment for Dioclea burkartii

XML Treatment for Dioclea fimbriata

XML Treatment for Dioclea guianensis

XML Treatment for Dioclea holtiana

XML Treatment for Dioclea lasiophylla

XML Treatment for Dioclea lehmannii

XML Treatment for Dioclea macrantha

XML Treatment for Dioclea ovalis

XML Treatment for Dioclea sericea

XML Treatment for Dioclea vallensis

XML Treatment for Dioclea virgata

XML Treatment for Dioclea virgata (Rich.) Amshoff var. virgata

XML Treatment for Dioclea virgata var. crenata

XML Treatment for Cymbosema

XML Treatment for Cymbosema roseum

XML Treatment for Cleobulia

XML Treatment for Cleobulia coccinea

XML Treatment for Cleobulia crassistyla

XML Treatment for Cleobulia diocleoides

XML Treatment for Cleobulia leiantha

XML Treatment for Macropsychanthus

XML Treatment for Macropsychanthus Harms subg. Macropsychanthus

XML Treatment for Macropsychanthus apiculatus

XML Treatment for Macropsychanthus aureus

XML Treatment for Macropsychanthus carolinensis

XML Treatment for Macropsychanthus circinatus

XML Treatment for Macropsychanthus comosus

XML Treatment for Macropsychanthus densiflorus

XML Treatment for Macropsychanthus dictyoneurus

XML Treatment for Macropsychanthus dolichobotrys

XML Treatment for Macropsychanthus edulis

XML Treatment for Macropsychanthus ferrugineus

XML Treatment for Macropsychanthus flexuosus

XML Treatment for Macropsychanthus funalis

XML Treatment for Macropsychanthus glabrus

XML Treatment for Macropsychanthus grandiflorus

XML Treatment for Macropsychanthus grandistipulus

XML Treatment for Macropsychanthus haughtii

XML Treatment for Macropsychanthus hexander

XML Treatment for Macropsychanthus huberi

XML Treatment for Macropsychanthus javanicus

XML Treatment for Macropsychanthus jamesonii

XML Treatment for Macropsychanthus latifolius

XML Treatment for Macropsychanthus lauterbachii

XML Treatment for Macropsychanthus lauterbachii

XML Treatment for Macropsychanthus lauterbachii

XML Treatment for Macropsychanthus lauterbachii

XML Treatment for Macropsychanthus malacocarpus

XML Treatment for Macropsychanthus marginatus

XML Treatment for Macropsychanthus megacarpus

XML Treatment for Macropsychanthus mindanaensis

XML Treatment for Macropsychanthus mollicomus

XML Treatment for Macropsychanthus pulchrus

XML Treatment for Macropsychanthus purpureus

XML Treatment for Macropsychanthus rufescens

XML Treatment for Macropsychanthus schimpffii

XML Treatment for Macropsychanthus schottii

XML Treatment for Macropsychanthus sclerocarpus

XML Treatment for Macropsychanthus ucayalinus

XML Treatment for Macropsychanthus umbrinus

XML Treatment for Macropsychanthus violaceus

XML Treatment for Macropsychanthus wilsonii

XML Treatment for Macropsychanthus

XML Treatment for Macropsychanthus bicolor

XML Treatment for Macropsychanthus coriaceus

XML Treatment for Macropsychanthus duckei

XML Treatment for Macropsychanthus erectus

XML Treatment for Macropsychanthus hispidimarginatus

XML Treatment for Macropsychanthus macrocarpus

XML Treatment for Macropsychanthus rigidus

XML Treatment for Macropsychanthus ruddiae

XML Treatment for Macropsychanthus scabrus

XML Treatment for Macropsychanthus scabrus

XML Treatment for Macropsychanthus scabrus

XML Treatment for Macropsychanthus scabrus

## Supplementary materials

Supplementary material 1 Figure S1 Data type molecular data Explanation note Bayesian 50% consensus cladogram and respective phylogram of the *Dioclea* clade resulting from the ETS analysis. Bayesian posterior probabilities are reported above branches and parsimony (left) and maximum likelihood (right) bootstrap support values are reported below branches. Bootstrap values below 50% are represented by hyphens. https://binary.pensoft.net/file/465934This dataset is made available under the Open Database License (http://opendatacommons.org/licenses/odbl/1.0/). The Open Database License (ODbL) is a license agreement intended to allow users to freely share, modify, and use this Dataset while maintaining this same freedom for others, provided that the original source and author(s) are credited.Luciano Paganucci de Queiroz, Cristiane Snak

Supplementary material 2 Figure S2 Data type molecular data Explanation note Bayesian 50% consensus cladogram and respective phylogram of the *Dioclea* clade resulting from the ITS analysis. Bayesian posterior probabilities are reported above branches and parsimony (left) and maximum likelihood (right) bootstrap support values are reported below branches. Bootstrap values below 50% are represented by hyphens. https://binary.pensoft.net/file/465935This dataset is made available under the Open Database License (http://opendatacommons.org/licenses/odbl/1.0/). The Open Database License (ODbL) is a license agreement intended to allow users to freely share, modify, and use this Dataset while maintaining this same freedom for others, provided that the original source and author(s) are credited.Luciano Paganucci de Queiroz, Cristiane Snak

Supplementary material 3 Figure S3 Data type molecular data Explanation note Bayesian 50% consensus cladogram and respective phylogram of the *Dioclea* clade resulting from the tnrK/matK analysis. Bayesian posterior probabilities are reported above branches and parsimony (left) and maximum likelihood (right) bootstrap support values are reported below branches. Bootstrap values below 50% are represented by hyphens. https://binary.pensoft.net/file/465936This dataset is made available under the Open Database License (http://opendatacommons.org/licenses/odbl/1.0/). The Open Database License (ODbL) is a license agreement intended to allow users to freely share, modify, and use this Dataset while maintaining this same freedom for others, provided that the original source and author(s) are credited.Luciano Paganucci de Queiroz, Cristiane Snak
