# Supplementary figures and images for: Revisiting the taxonomy of Dioclea and related genera (Leguminosae, Papilionoideae), with new generic circumscriptions
Source: PhytoKeys. 2020 Oct 21;164:67–114. doi: 10.3897/phytokeys.164.55441 (PMC8359005; doi:10.3897/phytokeys.164.55441)

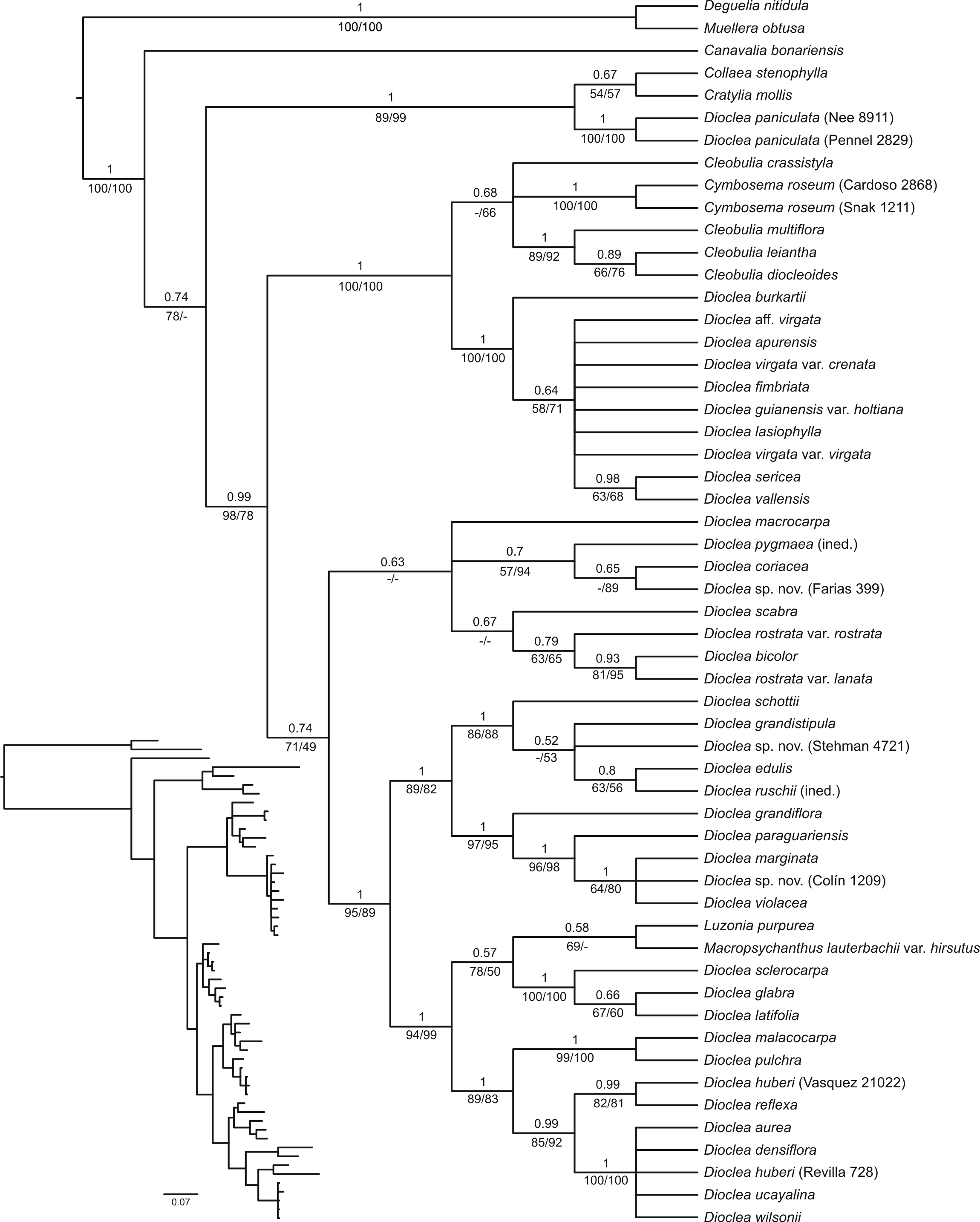

Supplement: Supplementary material 1 — Figure S1 [file phytokeys-164-067-s001.tif]

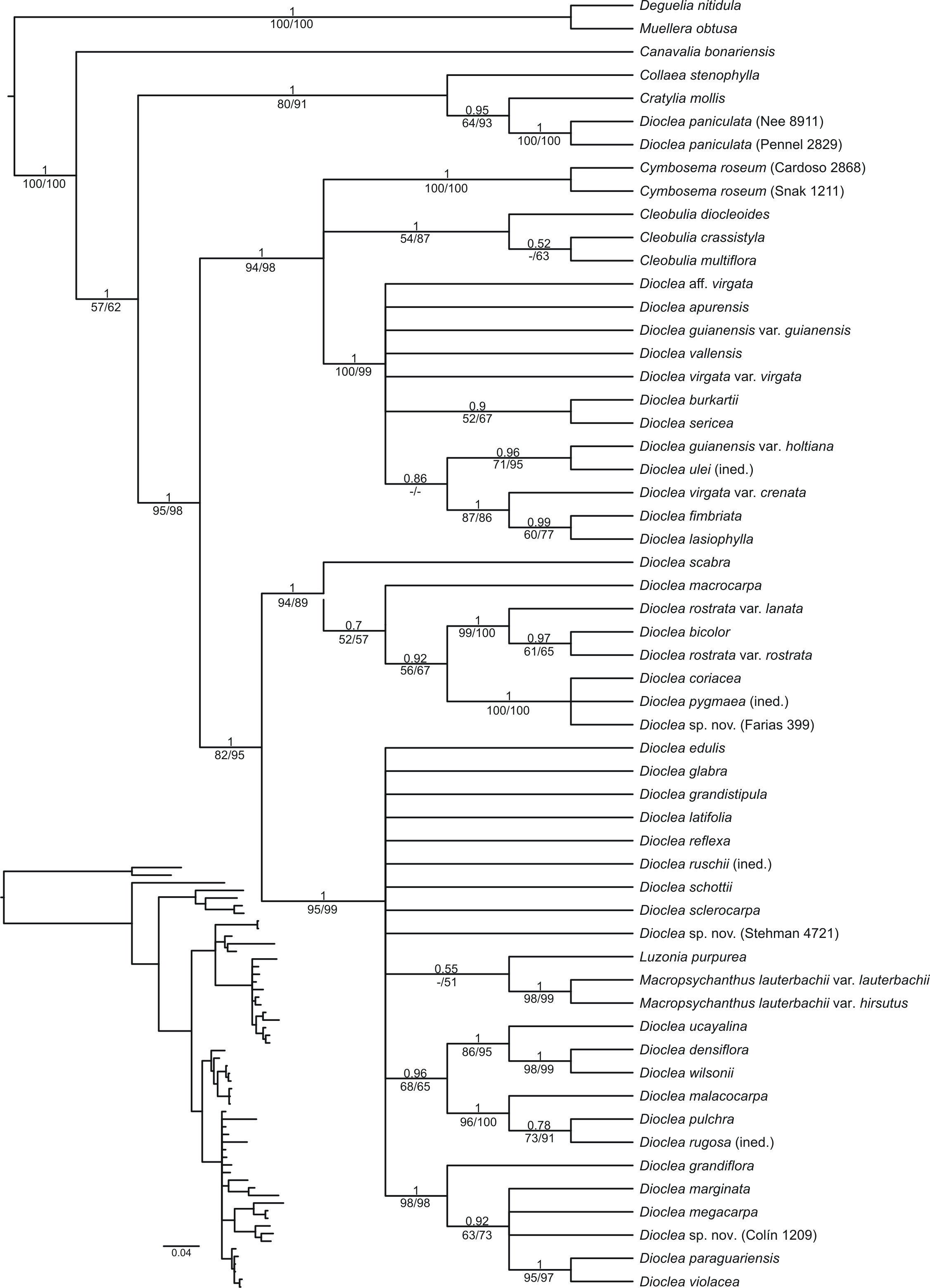

Supplement: Supplementary material 2 — Figure S2 [file phytokeys-164-067-s002.tif]

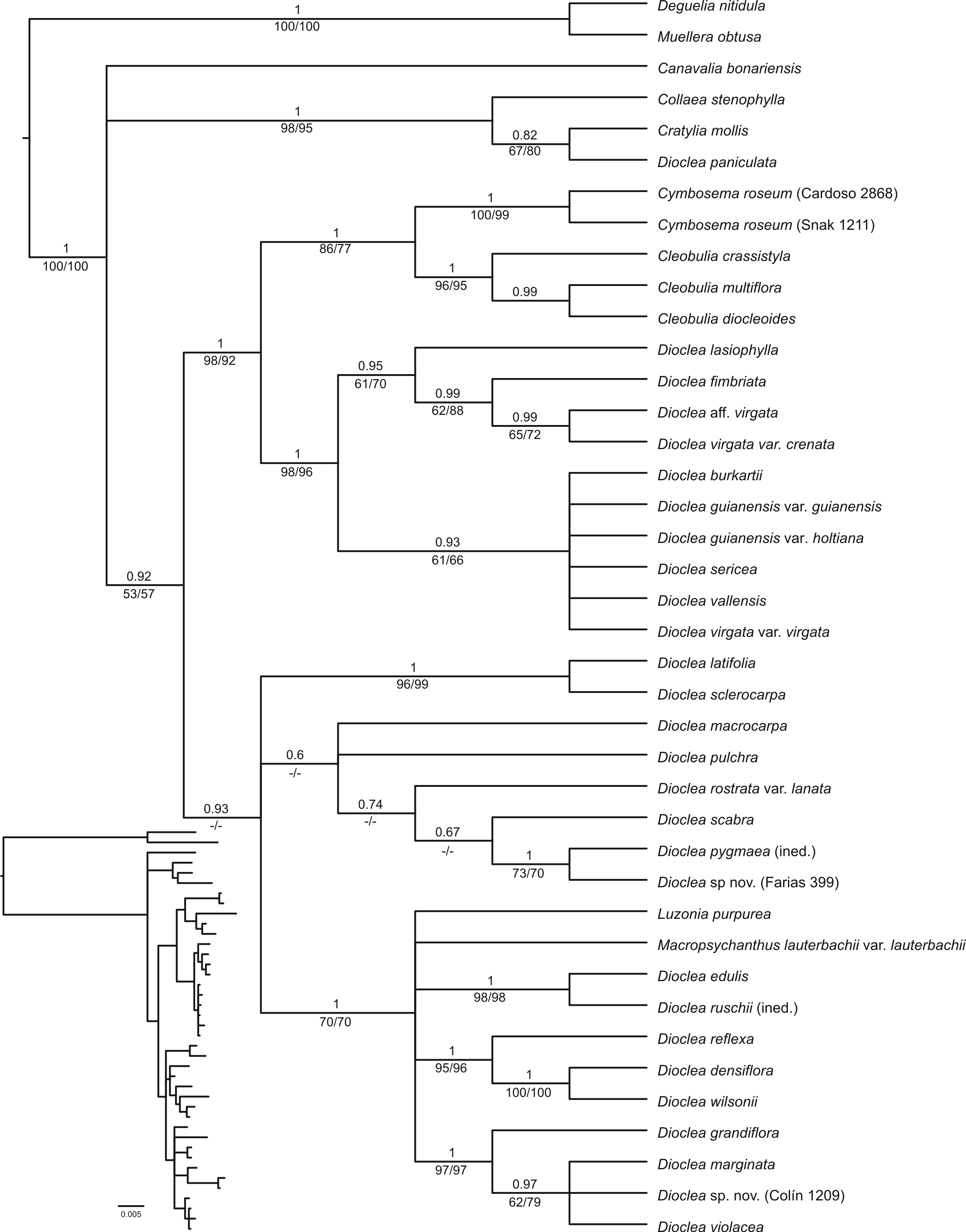

Supplement: Supplementary material 3 — Figure S3 [file phytokeys-164-067-s003.tif]
